# Emerging Themes in CryoEM—Single Particle Analysis Image Processing

**DOI:** 10.1021/acs.chemrev.1c00850

**Published:** 2022-07-04

**Authors:** Jose Luis Vilas, Jose Maria Carazo, Carlos Oscar S. Sorzano

**Affiliations:** Biocomputing Unit, Centro Nacional de Biotecnologia (CNB-CSIC), Darwin, 3, Campus Universidad Autonoma, 28049 Cantoblanco, Madrid, Spain

## Abstract

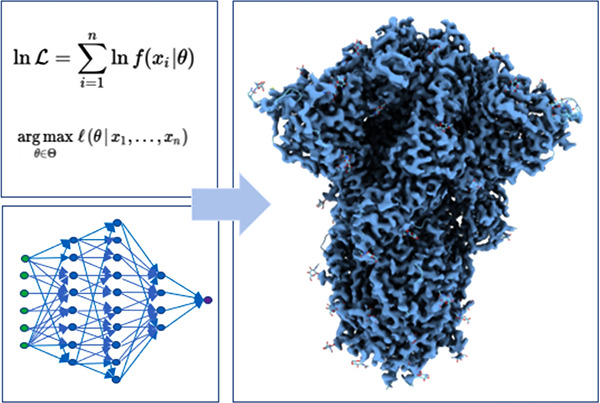

Cryo-electron microscopy (CryoEM) has become a vital technique in structural biology. It is an interdisciplinary field that takes advantage of advances in biochemistry, physics, and image processing, among other disciplines. Innovations in these three basic pillars have contributed to the boosting of CryoEM in the past decade. This work reviews the main contributions in image processing to the current reconstruction workflow of single particle analysis (SPA) by CryoEM. Our review emphasizes the time evolution of the algorithms across the different steps of the workflow differentiating between two groups of approaches: analytical methods and deep learning algorithms. We present an analysis of the current state of the art. Finally, we discuss the emerging problems and challenges still to be addressed in the evolution of CryoEM image processing methods in SPA.

## 1 Introduction

**Figure 1:**
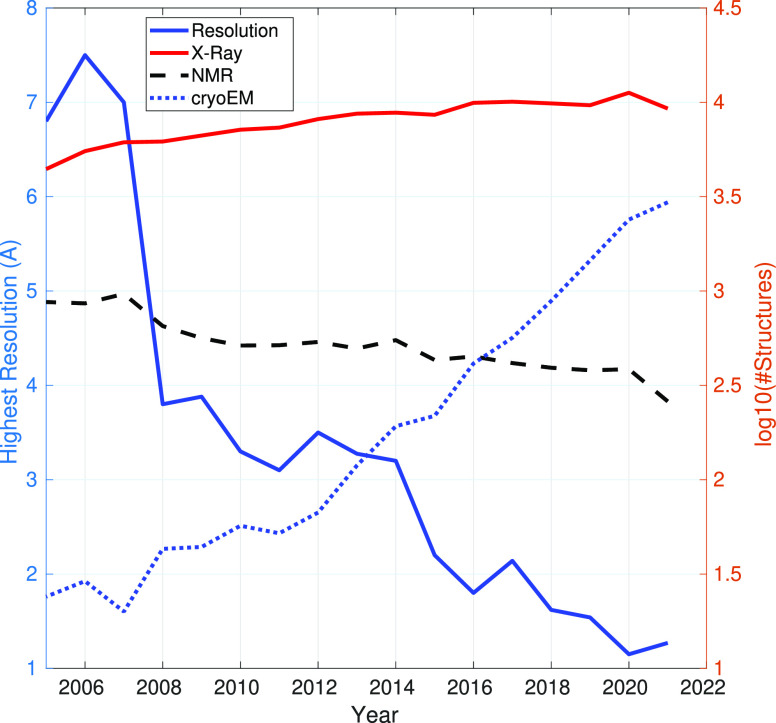
Yearly evolution of (left axis) the highest resolution achieved by CryoEM and (right axis) the number of deposited structures in the PDB by experimental method. Data extracted from ref (16).

Structural biology aims to elucidate the three-dimensional structure of biological macromolecules to understand their working mechanisms in physiological and pathological contexts. The applications are multiple, from developing new drugs to designing proteins for carrying out specific tasks. Indeed, the field has witnessed a swift expansion in the past decade. The number of new structures deposited each year into databases such as the Protein Data Bank (PDB)^16^ proves its impact. To have a quantitative understanding of the quick growth of the field, we mention that the first atomic models were determined in the decade from 1960 to 1970, but currently, there are more than 180,000 structures^16^ in the PDB. The main experimental techniques contributing to this growth are X-ray crystallography, nuclear magnetic resonance (NMR), and, more recently, cryo-electron microscopy (CryoEM). Looking at the statistics on the PDB website, the X-ray crystallography method is responsible for most of the database entries. However, the increase of models solved by CryoEM has steadily grown in the last ten years (see Figure 1), making it the fastest-growing approach. The relatively steep rise of CryoEM over other structural techniques is mainly due to its capacity to study biological entities in close to physiological states with reduced requirements in terms of sample quantity and concentration and without crystallization needs.

At present, the most common CryoEM techniques are (1) single particle analysis (SPA), which considers a purified sample containing multiple copies of the purified protein under study, (2) electron tomography, which works with “in situ” preparations (i.e., without many purification steps which could potentially disturb the structure, allowing the direct study of the cellular environment in its native state and pushing the understanding of protein interactions, and (3) microelectron diffraction, probably the “newest branch”, offering some unique opportunities in those cases in which microcrystals are available, from small molecules to membrane proteins.^41^

Among them, SPA has probably been the main driver of the current widespread recognition and impact of CryoEM. To achieve this broad success, many challenges have had to be solved, such as developing a new generation of detectors, enhancing the electron optics and associated instrumentation, developing new vitrification techniques, and, very importantly, radically transforming new software and image processing methods.^210^ In 2014 most of these problems were adequately addressed, and resolutions close to 3 Å were obtained for the first time for noncrystalline specimens (see Figure 1); nowadays, that resolution is very often obtained. It is not then surprising that this trend was then called the *resolution revolution*(83) and the technique received the “Method of the Year 2015” award^100^ from *Nature Methods*. The impact of these advances on the structural biology community and, more generally, on the understanding of biological macromolecules was so high that, two years later, Jacques Dubochet, Richard Henderson, and Joachim Frank were awarded the Nobel prize in Chemistry for developing CryoEM “for the high-resolution structure determination of biomolecules in solution”.^194^

The future of this technique looks very promising, making a review with a focus on recent image processing methodologies and workflows in CryoEM single particle analysis particularly pertinent. In this way, we will analyze how we have reached the current situation of the field and what is considered state of the art for the different processing steps. Finally, we will take the opportunity to discuss those emerging topics and technologies that, in our opinion, will define the next steps forward in CryoEM. We have focused our review on the methodological developments performed over the past decade. CryoEM is a multidisciplinary field; this review attempts to cover the point of view of image processing. However, the reader can take the benefit of many other complementary reviews: about specimen preparation,^2^ about membrane proteins,^84^ about structural studies,^109^ about CryoEM limitations,^34,47,91^ about emerging issues,^210,218^ or about computational methods.^162,207^

## 2 Brief Introduction to Image Processing Approaches

The continuous advances in computational capabilities have allowed for an enormous revolution in data analysis and big data. Indeed, image processing in CryoEM is all about these two technologies. To have a coarse idea of the computational problem, consider the usual numbers associated with a typical CryoEM project. The fast acquisition rate of current detectors allows acquiring movies of the sample composed of many frames; the sum of the frames results in an image called a *micrograph*. In a normal microscopy session, hundreds or thousands of movies are acquired. Each movie has around 60 frames (depending on the dose) with dimensions of at least 4000 × 4000 pixels (and often more). Thus, raw data acquired by the microscope can easily be measured in terabytes. In these images, the individual macromolecules of interest need to be located and cropped from the micrographs. The number of cropped images (particles) can go from several hundred thousands to several millions. Assuming that the typical dimensions for each of these particles are 300 × 300 pixels, then the number of collected pixels is on the order of 300 × 300 × 2*M* = 18 × 10^10^ and the reconstructed structure will be a volume of 300 × 300 × 300 = 27 × 10^6^ voxels.^172^ These numbers mean that we want to solve a problem involving millions of unknown variables and thousands of millions of equations. This is a big data problem in a huge dimensional space, a difficult problem that gets further complicated by the very low signal-to-noise ratio (SNR) of CryoEM images and the intrinsic heterogeneity of the sample. Thus, image processing in CryoEM represents a real algorithmic and computational challenge.

The SPA image processing workflow is divided into smaller steps to solve the overall problem of reconstructing a biological macromolecule. There exists a wide variety of mathematical methods to undertake these tasks. In an effort to organize this varied information, in this review we will classify methods into two groups: analytical and deep learning methods. Traditionally, SPA has used classic image processing approaches based on what we will call *analytical methods*. In this group, we can find many different algorithms; Bayesian and regularization methods are good representatives. However, the recent coupling of the routine experimental collection of enormous data sets, the advent of new algorithms, and increased computational capabilities have resulted in what we can call the “data revolution”. This scenario is very well suited to approaches referred to as *deep learning methods* because they require high computational capabilities and large data sets to train.

### 2.1 Analytical Methods

This group considers all methods that can be formulated in direct mathematical terms, a characteristic that separates them from the deep learning approaches that will be presented in the next section. However, since many concepts are common to both analytical and deep learning methods, throughout this first section we will briefly point to some of their similarities and differences, always aiming at providing the reader with a broader perspective on the different approaches available for data analysis.

Algorithms, in general, can be of a very different nature. Still, at their core, there is usually some kind of evaluation of the similarity or dissimilarity of two images (correlation, distance between images, such as the Euclidean or any other distance). This similarity comparison is generally referred to as the data fidelity term. In its most common Euclidean formulation, it takes the form
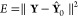
1where **Y** is a vector of observations and  is a vector of predicted values. This very generic formulation applies to many subproblems along the image processing pipeline. For instance, in 2D classification, **Y** is the experimental projection of a single particle, and  is the 2D class representative. We can further decompose this model into smaller pieces. For instance, let us assume that we predict that there is a clean image **X**_*i*_, called the class representative, that is affected by the microscope’s contrast transfer function (CTF), denoted by *C*, and then reoriented with an operator *A* to fit the orientation of the experimental image. Under this model, our prediction with this class representative would be . We would assign the image **Y** to the *i*-th 2D class that minimizes the energy of the error *E*, understanding the energy of the error as the square of the Euclidean distance . In fact, it can be easily shown that minimizing the error energy and maximizing the correlation between **Y** and  are equivalent under certain, but rather general, circumstances.

(**Side Note**: Let us consider, for instance, the problem of finding the geometric transformation, *A*, that minimizes the error between an observed image **Y** and the transformed prediction *A***X** =  = . Let us assume that *A* is applied in such a way that ∥*A***X**∥^2^ = ∥**X**∥^2^. This is true if we use image wrapping during the geometrical transformation, as is done, for instance, by the discrete Fourier transform, and the absolute value of the determinant of *A* is 1, as is the case for a rigid transformation. Then, ∥*A***X**∥^2^ does not depend on *A*, and the transformation that minimizes the error is the same as the one that maximizes the dot product between the two signals ( = . Let us consider now the cross-correlation between the two signals, , where  denotes a vector of the same size as **Y** with all its components set to the average of **Y**. If we use wrapping, then the average of *A***X** is the same as the one of **X** and its energy does not change either. Consequently, we may remove from the maximization of the correlation all the terms that do not depend on *A*, . Because all the components of  are equal, the term  is proportional to the mean of *A***X**, which does not depend on *A* due to the wrapping and can also be eliminated from the optimization. Finally, we get that the geometrical transformation that maximizes the correlation, , is the same as the one that minimizes the Euclidean distance. This result also holds in Fourier space with complex components, as long as each Fourier component has the same weight in the Euclidean distance calculation.)

Minimizing the Euclidean distance between two vectors may seem a very natural objective. However, this action has a critical statistical interpretation. If the data is supposed to be generated by an additive model of a deterministic underlying signal, **Y**_0_, plus (random) noise, **N**,
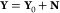
then, we wonder which is the estimate of **Y**_0_ that maximizes the likelihood of observing a particular realization of the random vector (a vector with random variables as components)
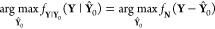
2where  is the conditional probability density function of observing **Y** given  and *f*_**N**_ is the probability density function of the noise. These probability density functions are also called likelihoods. For this reason, our best estimate, , is called the maximum likelihood solution.

It is customary to maximize the logarithm of the likelihood, instead of the likelihood,
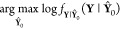
Because the logarithm is a monotonic function, the location of the maximum of the likelihood is the same as the location of the maximum of the log-likelihood. The reason for this transformation is that when we analyze many realizations of the same random vector, , we want to optimize the model that maximizes the likelihood of the set, not of any image in particular; the fact that we are dealing with logarithms makes this latter task particularly simple to express. Indeed, because the realizations are independent, this can be easily decomposed as
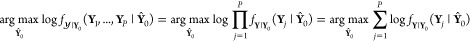
If we assume the noise, **N**, follows a multivariate Gaussian distribution centered at **0** and with covariance Σ,
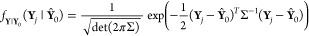
then the optimization of the log-likelihood above after removal of the terms that do not depend on  becomes
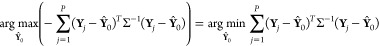


If we assume that all the components of the noise have the same variance, σ^2^, and that all of them are independent of each other, then Σ = σ^2^*I*, with *I* being the identity matrix of the same size as the length of the noise vector, and the optimization problem becomes
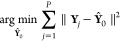


That is, we have come down to the Euclidean distance minimization with which we started this section.

From this small exercise, we may draw many important and general ideas:

1. Optimizations whose objective function is a data fidelity term can be understood as a maximum likelihood problem for some given statistical distribution of the noise.
2. Assuming a multivariate Gaussian distribution with equal variance and independent components for the noise (what is known as additive white Gaussian noise, AWGN) translates into an elementary least squares problem in which the goal function is just the Euclidean distance between the observations and our predictions.
3. All algorithms in CryoEM in which the cross-correlation between an experimental image and a reference is maximized, such as projection matching or the inner steps of most algorithms in which two images are aligned, are also maximum likelihood solutions of an image formation model in which we assume AWGN.
4. In the CryoEM field, it is normally assumed that projection matching and maximum likelihood methods are two families of solutions with different properties. This understanding stems from the specific way the two methods were introduced to the field. In this way, in classical CryoEM projection matching^115^ the estimate  is searched as the maximally correlating image between the experimental image **Y** and a collection of reference images, with the search for the maximum having a non-negligible chance of being trapped in a local optimum. In classical CryoEM maximum likelihood,^148,149,161^ in turn, the estimate  is calculated as a weighted sum of all the reference images with different weights (computed from a likelihood reasoning). However, the latter approach to maximum likelihood is the result of applying a particular (and very successful) algorithm to solve maximum likelihood problems called expectation-maximization, which in itself could have been solved differently using gradient descent or any other approach. The advantage of expectation-maximization is that it can handle latent, unobserved variables (for instance, in the CryoEM field, the angular assignment is treated as unobserved variables that must be marginalized). In much the same way, projection matching could have been implemented, at least conceptually, as an exhaustive search for the optimal value. So, we are always solving maximum likelihood problems, and the difference is the way this optimization is performed. In short, expectation-maximization has proven to be a compelling optimization technique in CryoEM. It opened the field to high resolution under the typically very high dimensional, very high noise conditions of cryogenic image acquisition without staining.
5. Assuming other statistical distributions translates into different optimization problems. For instance, a general multivariate Gaussian distribution for the noise with arbitrary covariance matrix Σ would result in a weighted least squares problem. The data fidelity term of RELION,^145^ formulated in Fourier space with different variances for each frequency, belongs to this family. Another example would be if instead of assuming a Gaussian distribution, we assume a Laplacian distribution, then instead of an Euclidean norm minimization, *l*_2_, we would have a *l*_1_ minimization. Conversely, given any data fidelity term, such as the Huber loss function or the correntropy used in CL2D,^166^ we could always construct a likelihood function whose logarithm is related to the term we are optimizing, even if this likelihood function does not have any known name (Gaussian, Laplacian, ...).
6. The maximum likelihood framework has been presented in an extremely generic way. All the steps we encounter along the image processing pipeline in CryoEM (movie alignment, CTF determination, particle picking and identification, 2D classification, 3D classification, volume restoration, ...) can be formulated in this framework. For each one of the problems, the roles of **Y** and  are played by different types of data and models.

The data analysis problems we have presented so far fall into a category called unsupervised data analysis problems. In this kind of problems, we are given a set of observations, **Y** vectors, and our goal is to make some sense of them. The most prominent example in CryoEM would be the 2D or 3D classification of the experimental images. Although we use the word classification in our field, a more technically correct word would be clustering: images are grouped because they all belong to the same conformational state, point of view, or any other feature of interest that is not explicitly stated. As opposed to unsupervised problems, another important branch of analysis is supervised problems. In this second branch, each observed vector is accompanied by a label that characterizes that observation. For instance, when we manually select particles in a micrograph, we attach a discrete label to each image patch of the micrograph so as to indicate whether that patch contains a particle at the center of the patch (representing this presence as 1, for example) or not (encoded as 0, for example). Loosely speaking, we can define a label as a number that identifies a given feature of the data. Classes can be created by the set of data with the same labels (identifiers). In this way, data now comes in pairs of observations and labels. The most common notation is to keep **X**_*j*_ for the observed vector, called the predictor variables, and **Y**_*j*_ for the label, called the predicted variables. We can attach more than one label to each observation (for instance, whether the image patch contains a centered particle and the kind of particle), and labels can be discrete or continuous. The goal of supervised data analysis problems is to find a function that helps us optimally predict the labels from the observations . If the predicted label is continuous, then the problem is called a regression problem. If the label is discrete, then the problem is called a classification problem. In CryoEM, we do not have a real 3D classification problem because we do not know the class labels for the experimental images. Classification problems are often formulated in a regression framework due to the more efficient optimization tools encountered for continuous variables. In this way, instead of just predicting 0 or 1 for a given image, we output a continuous value between 0 and 1, indicating our belief in whether the given image is more likely (closer to 1) or less likely (closer to 0) to contain a particle in the middle. One of the most famous transformations of a classification into a regression problem is the logistic regression, which is at the core and output of many deep learning formulations.

Regression problems can also be set in the maximum likelihood framework. Let us consider a family of functions *f*_Θ_(**X**_*j*_) defined by a set of parameters Θ (for instance, the family of functions *y* = *f*_*a*,*b*_(*x*) = *a* + *bx* is defined by the parameters *a* and *b*). Given a set of (**X**_*j*_, **Y**_*j*_) pairs, we look for the parameters that better allow us to perform the predictions
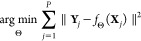
3Interestingly, this is exactly the kind of optimization problem solved by deep learning methods. In that regard, the data fidelity term of neural networks can also be considered a maximum likelihood regression problem. Notably, the function *f*_Θ_ in the deep learning setup is much more sophisticated and powerful than functions used in standard regression problems. The optimization algorithms to find Θ are also much more robust in deep learning since they have to deal with the drawback of performing the optimization in a very high dimensional space (the size of the vector Θ is very large, as we will see in the next section). Finally, the loss function (coarsely speaking error) in deep learning is not restricted to the Euclidean distance between the observed and predicted values, but the field has explored a vibrant landscape of possible loss functions.

Related to this regression formulation is the one of inverse problems, greatly advocated for in CryoEM. We could formulate the problem as
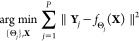
Note that we are given a set of observations, **Y**_*j*_, and we must find the parameters, Θ_*j*_, and a single predictor, **X**, that explains our observations. This would be the case of 3D reconstruction in CryoEM. For each experimental image, **Y**_*j*_, we must find some 3D alignment parameters, Θ_*j*_, and a volume **X** such that when we project the volume along the direction given by Θ_*j*_, that is, , this reprojection looks as similar as possible to the observation.

The last kind of problems encountered in CryoEM and addressed here is the autoencoding approach. For each experimental observation, we must find the set of parameters that best explains that observation:
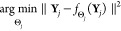
This problem is restricted to construct an internal representation whose dimension is smaller than the dimension of the vector **Y**_*j*_. In this way, the trivial identity solution does not belong to the family of functions .

A CryoEM problem of this class would be the determination of the defocus: for each micrograph, we would calculate its power spectral density (PSD), **Y**_*j*_, and we must find the defoci, Θ_*j*_, that best explain that PSD. Note that the autoencoding is performed in an inherently parallel fashion; that is, the determination of the defoci of one micrograph is independent of the defoci of any other micrograph, and that is why there is no sum over *j* in the objective function. Interestingly, autoencoding is a widespread strategy in deep learning due to the common absence of labels attached to each observed image. In deep learning autoencoders, the function *f* depends on a set of parameters specific to each observation but also on a set of common parameters, , that are optimized as well,
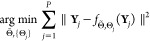


Therefore, the problem cannot be formulated now as a collection of *P* independent subproblems, but the whole set must contribute to determining the function parameters.

As we have seen, data fidelity terms are related to a maximum likelihood formulation and finding the model that makes the observations maximally likely. However, we may extend this framework by incorporating *a priori* knowledge of the atomic models through another probability density function, in this case, of the models . In this way, the new setup comes after a Bayesian formulation of the problem (see eq 2 for its maximum likelihood counterpart)
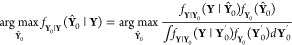
where the denominator is *f*_**Y**_(**Y**). Because  is its integration variable, the denominator does not depend on our choice of . Consequently, it can be eliminated from the optimization. As we have a product of likelihoods, it is also convenient to take its logarithm, and because this transformation is monotonic, the best model, , will still be the same. Our optimization problem now is referred to as maximum *a posteriori* (MAP), and it is formulated as
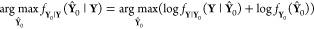


The first term is the data fidelity term that we already studied in the discussion on the maximum likelihood. The second term comes from our prior information on the set of models we are looking for. As we did with the maximum likelihood, we may assume a particular distribution for this term, for instance, a multivariate Gaussian distribution with equal variance, zero mean, and independent components. As we saw in the part related to maximum likelihood, this assumption, after removing all the terms that do not depend on , results in a term of the form
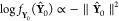
where ∝ denotes proportional to. This is the famous Tikhonov regularization in regression problems. The prior of RELION belongs to this family. Priors are great to use if they truly correspond to reality. Unfortunately, this easy-to-handle multivariate Gaussian prior for the model is not strictly verified by macromolecules,^178^ and consequently, its use necessarily biases the results. However, it is well-known in the statistical literature that when the number of observations is huge, as in CryoEM, the data fidelity term is much larger than the term coming from the prior, and it dominates the maximization process. The reason is that the data fidelity term grows with the number of observations (it depends on **Y**), while the penalization term does not.

In regression, it is common to formulate the problem of combining data fidelity and *a priori* knowledge (or “constraints” or “penalization term”) through a weighted combination of these two terms that makes a new expression. We commonly refer to this process as regularization, and it yields expressions of the form
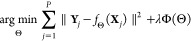
where λ is a constant that balances the weight between the data fidelity term and the penalization term Φ(Θ), for some positive function Φ called regularizer or penalization term. Even λ may have an interesting statistical interpretation. For example, for Tikhonov regularization and a least squares fidelity term, it is easy to show that  (where  and  are the variances of the noise and the model components, respectively). That is, λ is the inverse of the signal-to-noise ratio (SNR). In this way, if the SNR is high, we will reduce the weight of the prior term with respect to the data fidelity term. Conversely, if the SNR is low, we will put more weight on the prior information.

Seeing the MAP formulation, we can always understand the regularizer as related to the prior distribution of the models. We have already seen that Tikhonov regularization comes from a multivariate Gaussian prior with zero mean, equal variance, and independent components. Other regularizations such as total variation (the *l*_1_ norm of the spatial gradient of the model, Φ(Θ) = ∥∇Θ∥_1_) can be understood as a Laplacian prior on the spatial gradient of the model. It is well-known that this prior promotes sparsity of the spatial derivatives of the model, which is good for lowering the noise in the estimated model. Still, it may not be necessarily true for biological structures. In general, we can always construct a prior distribution whose logarithm results in the regularizer we use.

The optimization strategy may vary. We may try to decrease the whole objective function simultaneously, as is done in RELION or deep learning approaches. In turn, we may alternate between steps that first minimize the data fidelity term and other steps that minimize the penalization term, as is done in ref (35). In this second alternative it is common to use proximity operators to increase the current solution’s prior likelihood. Among those most used, we encounter the soft-or hard-shrinkage operators used with *l*_1_ norms.^81^

Additionally, we may employ what is referred to as noninformative priors. In this case, we assign the same probability to all feasible solutions. For instance, we may know that our solutions have to be non-negative. However, any model, , fulfilling this condition is equally likely. Projection onto convex sets^20,179^ could be seen as a proximity operator that chooses one of the possible solutions given by the uninformative prior and our current model estimate. Following this reasoning, a pure maximum likelihood problem can be seen as a MAP problem in which all solutions are equally likely. Consequently, the prior likelihood does not depend on our specific choice of model, , and it is eliminated from the goal function.

From the digression above, we may draw the following conclusions:

1. Any image processing step we perform along the CryoEM image processing pipeline can be understood in a MAP framework with the appropriate choice of probability density functions: one for the distribution of the noise that gives rise to the data fidelity term and another one for the kind of models we are looking for that gives rise to the penalization term.
2. We may choose priors that can be easily handled mathematically, although they may not represent real priors of biological macromolecules, or we may choose priors that faithfully represent biological features but may then be much more difficult to deal with mathematically (and computationally). A certain equilibrium is always needed.
3. Deep learning algorithms are not inherently different from the more classical algorithms with respect to the general MAP setup in which they can be formulated. However, they are inherently different in the complexity of the functions *f*_Θ_ being sought. Consequently, they require completely different optimization algorithms and much more data to be able to faithfully estimate the large number of parameters required.

In this section, we have focused on the kind of optimization problems being solved in CryoEM. We have put them into a single generic framework (MAP) and subsequently analyzed the specific choices made by the different algorithms used in CryoEM. Underneath this apparent similarity at a high level, we must now consider that there are significant differences in the implementation of the algorithm, the optimizer, the choice of initial values, and many numerical tricks, such as not evaluating the full probability density function but only a part of it exploiting the relationship between Fourier and real space, etc. These differences make important distinctions between the various algorithms and result in different properties concerning robustness to noise, convergence speed, resilience to perturbations of the algorithm initialization, wall-clock execution time, memory requirements, etc.

An important idea to keep in mind is that any image processing algorithm in CryoEM is about the estimation of some underlying model  (local frame displacements, defocus values, particle locations, 2D or 3D classes, the angular orientation of each particle, or any other parameter or model we may think of). Because the input data is random (because of the noise), the estimate of the underlying model  is another random variable. Because the SNR of the input images is so low, between 0.1 and 0.01, the variability of the estimated parameters may be quite large. In general, given this situation, the best approach we can have (that is, however, seldom done in CryoEM) is to estimate each parameter multiple times, ideally using algorithms with different rationales and mathematics behind them, after which we compare the different estimates. If they are sufficiently close, the average of these estimates will surely have a lower variance. If they are sufficiently apart, we need clustering to identify the most likely region for the ground-truth model for that particular input. This clustering approach can be taken if there are at least three independent estimates. If there are only two and they disagree, the most we can do is discard this input image as we cannot be sure which of the two is the correct estimate of the underlying model. The interested reader may consult ref (168) for an expanded discussion of bias and variance in the estimation of parameters in CryoEM and how what we normally call overfitting is caused by bias in the estimation of the parameters.

### 2.2 Deep Learning

As we have seen, the objective functions of any deep learning algorithm can be understood in the context of either a maximum likelihood or maximum *a posteriori* formulation. In general, they are known as loss functions in the deep learning literature. There has been a very active search of various possibilities^86,227^ beyond the Euclidean distance presented in the previous section.

Conceptually speaking, deep learning algorithms are solving nothing more than regression, classification, or autoencoding problems with nonlinear functions, *f*_Θ_. This goal is shared with more traditional image processing or machine learning algorithms. Historically, they inherit from a tradition of neural network algorithms that had their first wave in the 1980s with a relatively important impact only in some niche applications. However, in the 2000s, they acquired the adjective “deep”, which we will explain later, and since then, they have become ubiquitous in most data and image analysis tasks. This leap was caused by several contributions that more or less coincided in time (for a review, see ref (151)):

- The number of network parameters was largely increased (by several orders of magnitude). Increasing the number of parameters obviously allows us to express much richer functions, but they are, in principle, much more prone to overfitting.
- The risk of overfitting data was reduced by (1) the availability of large amounts of training data; (2) the exploration of nonlinear functions different from the sigmoids (such as the logistic function) traditionally used in the neural network field; (3) the strong reduction of parameters achieved by reusing them, with one of the first approaches being the introduction of convolutional neural networks (see more below); (4) improving the backpropagation of the gradient of the loss function; two prominent approaches are residual networks and batch normalization (see more on these below); and (5) the use of stochastic optimizers, that allowed reaching useful solutions (if not the global minimum of the goal function). In addition, there have been many more important advances, such as the development of attention^203^ and transformers,^33^ but they have not substantially reached CryoEM for the moment, and they will not be discussed further. Many efforts have been addressed to understand the learning mechanism of deep algorithms, and two important ideas seem to emerge:
  1. Only a small fraction of the network is actually useful to make the prediction. This has been known as the lottery ticket hypothesis.^40^ The idea is that having a large network with many randomly initialized weights “buys” many lottery tickets (subnetworks), and some of them will be optimized to learn the relationship between **X** and **Y**. For sufficiently wide networks, it seems that most local minima are close to the global minima and that the dangerous locations are not the local minima but the saddle points (points at which the loss function locally looks flat in most directions).^185^
  2. The *f*_Θ_ functions learned by deep learning are good interpolators in the high dimensional space of **X** but extremely bad extrapolators, even for **X** vectors whose appearance is not qualitatively different from the training data.^102^ This means that there can be catastrophic errors for test data that looks like the training data but whose location in the space of **X** is far from the location of the data used for training. Data augmentation operations such as image rotation, scaling, shifts, mirroring, adding noise, etc. and variational approaches (see more below) have been adopted to enlarge the space covered by the input training data.
- The exploitation of massively parallel hardware as provided by graphical processing units (GPUs). These hardware elements have a computational capability much higher than that of the general-purpose CPUs. The price to pay is that the program control flow must be rather linear, without too many branches or loops. However, deep learning problems can be conceptually cast into this class of executions, and the underlying libraries (such as Tensorflow or Pytorch) have been efficiently ported to GPU execution.

In its most basic formulation, a neuron is a nonlinear function of its inputs, *x*_*i*_, of the form
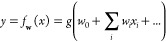
where *g*(·) is a nonlinear function called the activation function, and *w*_*i*_ are a set of real numbers to be set called weights. In other words, a neuron is an activation function . In fact, the activation function by itself can be understood as the optimal maximum *a posteriori* criterion that separates two classes depending on different assumptions regarding the distribution of the neuron inputs^42^ (for instance, the optimal separation of multivariate normal inputs into multiple classes is performed by a softmax activation function).

This family of functions started as a way to simulate the physiological behavior of biological neurons but quickly grew past this point. A neural network is an arbitrary composition of functions of this type; that is, it is the connection of many neurons such that the inputs of some neurons are the outputs of the others. As an example, and in the subfield of regression, a neural network (see Figure 3) is a universal approximator; that is, any sufficiently smooth function can be approximated to any degree of accuracy by a neural network of increasing complexity.^196^ Note that not all the activation functions of the network need to be the same. For instance, it is typical to use any of the ReLU (rectifier linear unit, defined as the function *ReLU*(*x*) = max(0, *x*)) variants in the hidden layers but to use in the output layer an identity (for regression problems) or a logistic one (for classification problems).

**Figure 2:**
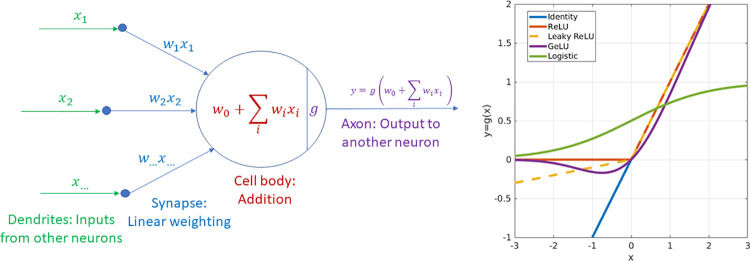
Scheme of a basic neuron: (left) Mathematically, it is composed by multiple inputs *x*_*i*_, which are linearly combined applying different weights, *w*. This linear combination is the argument of the activation function *g*(·). (right) Several common activation functions are shown.

**Figure 3:**
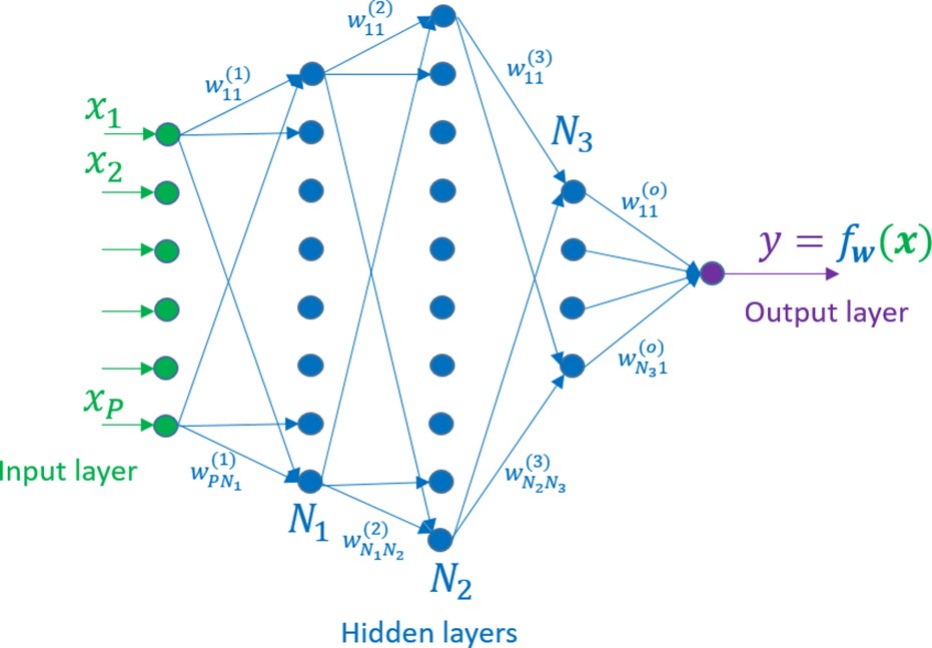
Feed-forward, dense neural network: the input signals *x*_*i*_ are propagated forward through neurons which are arranged in layers of *N*_*j*_ elements as in Figure 2 until they reach the output neuron. The weights *w*_*ij*_ control the propagation of the signal through the neurons and layers.

In their most basic form, a feed-forward, dense neural network predicts a value *y* from a set of inputs **x** by propagating forward the **x** values through a network of neurons such as the one shown in Figure 2 in which all neurons in one layer are connected to all neurons in the next layer. We normally distinguish between the input layer, the middle or hidden layers, and the output layer. The parameters of the function are the weights of each one of the connections. We can increase the complexity of the calculated function, to enhance the approximation that the network produces, by adding more hidden layers and move neurons in each layer. The adjective “deep” comes from the fact that there are many hidden layers (for instance, ResNet-50, one of the state-of-the-art networks to classify images, has 50 hidden layers). The total number of parameters for a dense network, such as the one shown in Figure 3, is (*P* + 1)*N*_1_ + (*N*_1_ + 1)*N*_2_ + (*N*_2_ + 1)*N*_3_ + (*N*_3_ + 1). The term +1 is because there are *P* input variables plus one that comes from the offset weight *w*_0_. These *P* variables are connected to *N*_1_ neurons of the first layer resulting in (*P* + 1)*N*_1_. When a second layer is added, the number elements of this one will be (*N*_1_ + 1)*N*_2_, again as a consequence of the offset weights. This is the origin of the total number of parameters (*P* + 1)*N*_1_ + (*N*_1_ + 1)*N*_2_ + (*N*_2_ + 1)*N*_3_ + (*N*_3_ + 1). As the number of layers and their complexity grow, the number of parameters of the function easily goes up rapidly. It is not uncommon to find networks with a few million parameters even for small applications (for instance, GPT-3, a neural network to process natural language, has 175 billion parameters).

**Figure 4:**
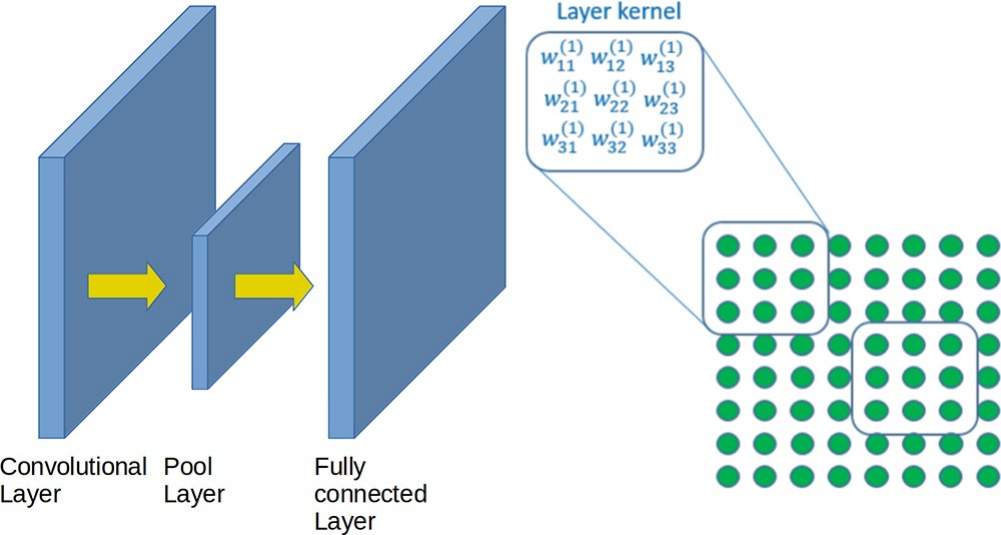
Example of a convolutional neural network. It is composed of a convolutional layer, followed by a pool layer, and ending in a fully connected layer. Each layer can be understood as a matrix of weights.

**Figure 5:**
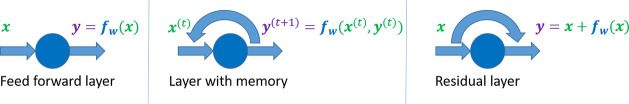
Examples of more advanced neurons considering different topologies depending on the presence of forward or backward skip connections.

An easy way to reduce the number of parameters is by reusing them. An image is particularly amenable to this because we will probably want to apply the same mathematical operation to an object in it, irrespective of whether it is placed on the left or the right of the image (see Figure 4). The same weights can be used for all input variables (pixels), regardless of their absolute position within the input image. Without the nonlinearity *g*, this operation is known as a convolution, which is why these networks are called convolutional. The weights used for the convolution are known as a kernel. In this way, the output of a single kernel applied to an input image is another image in which we have performed a convolution and applied a nonlinear function, *g*, to the convolution output. The number of parameters is reduced from (*P* + 1)*N*_1_, for the first layer, to *K*^2^ + 1, where *K* × *K* is the size of the kernel. For instance, for an image of size 512 × 512 (*P* = 512^2^), a hidden layer of the same amount of neurons (*N*_1_ = 512^2^), and a kernel of size 11 × 11, the number of parameters of the first layer, **w**^(1)^, decreases from (512^2^ + 1)512^2^ ≈ 69 × 10^9^ to 121. This is a decrease by 8 orders of magnitude. Because convolutional neural networks (CNNs) require so few parameters, we can afford to learn multiple kernels in the same layer. Now, the output of layer 1 will not be an image but a stack of images called a tensor. This possibility of learning multiple kernels within the same layer is represented in Figure 4 by stacking several images within the same layer. We may increase our artillery of nonlinear functions by introducing other operations. Among them, one of the most successful has been the max-pooling operation (taking the maximum value within a neighborhood), which is frequently found in image processing neural networks as it somehow keeps the most important (most energetic) features of the previous layer. In fact, we may be very creative here and introduce any nonlinear operation or network architecture as desired. For instance, we may calculate the output of a neuron based on its own value (then, we have neurons with memory), or we may force the neuron to calculate an output that looks like the input plus a small deformation, called a residual; see Figure 5. In general, we can generalize the concept of the neuron to any nonlinear computational block.

The obvious problem of neural networks is how to estimate the function parameters **w** to minimize the error between the prediction of the network, *f*_**w**_(**X**_*j*_), and the desired value **Y**_*j*_ (see eq 3). We need a mechanism to update the network weights so that the loss function is minimized. In general, this process is backpropagation of the loss function from the output layer to the input layer, updating in this process the network weights backward in such a way that the same input **x** would incur a smaller loss, ∥**y** – **f**_**w**_(**x**)∥. Arbitrary computing blocks particularly complicate this mechanism. The development of automatic differentiation algorithms has greatly simplified this task^13^ as they can calculate the dependence (derivative) of the loss function on each of the parameters in the network. The optimization of the loss function is performed through some variant of gradient descent. The gradient is calculated by the automatic differentiation module, and many variants have been proposed from stochastic gradient descent, adaptive gradient, etc., with all of them trying to escape the many local minima expected for such a high-dimensional optimization. For a review of the optimization algorithms used in deep learning, see ref (186).

Due to the large number of parameters of deep learning models, it can be expected that the optimization of their parameters can be rather difficult. Several tricks are employed when trying to find useful networks:^159^

- Adaptive learning rates: in any gradient descent algorithm, the learning rate indicates how much we must move from the current solution, **w**^(*t*)^, to the next one, **w**^(*t*+1)^, that is supposed to be better in terms of the loss function. A too-small learning rate makes the learning process too slow and prone to local minima. In contrast, a too-large learning rate may make the optimization unstable and prevent the identification of very narrow local minima. There has been much research on designing algorithms that automatically adapt the learning rate to the local gradient size.^136^
- Vanishing gradient: one of the main problems of backpropagation in deep networks is that error may quickly dissipate in the first few layers close to the output layer. Technically, this is called the vanishing gradient problem. Two techniques have been shown to have a strong impact against this problem: batch normalization and the use of residual layers (also called the addition of skip connections; see Figure 5). Both techniques have to be explicitly integrated into the network architecture. Batch normalization is a module that numerically recenters and rescales the output of the previous layer. Skip connections propagate the information at a given layer forward so that its energy is not lost throughout the network. Skip connections can also be thought of as an easy way to force the network to produce an output similar to its input except for a small difference, the residual, that has to be learned by the network.
- Weight initialization: Much work has been devoted to initializing the network parameters and how to relate this process to the network number of layers, neurons, etc. Weights are typically randomly initialized with zero mean and some variance. However, a judicious choice of this variance is crucial because the energy of the propagating signals may easily saturate the response of the internal neurons (function *g* in Figure 2), falling again into a vanishing gradient problem.
- Transfer learning: In problems in which the network cannot learn due to the initialization of the network weights or the lack of data, it is useful to fix some of the most costly layers in terms of parameters. With this aim, our network will be formed by some fixed layers that will not be optimized and some other layers that will be optimized. The fixed layers are pretrained for some other related problem. For instance, it is typical to use the weights and architecture of a network trained on ImageNet,^32^ a data set with 14 million natural images with labels about the content of the images (such as persons, computers, sunsets, dogs, ...) and then add extra layers to adapt the whole network to solve a problem in another domain. The idea behind this is that the first layers of a neural network tend to learn low-level features of the input domain, such as edges in different orientations in the case of input images. Then subsequent layers combine the outputs of the low-level layers into higher-level features (such as recognizing a dog). If we need to tackle a problem in CryoEM, in which, obviously, we do not have people or dogs in the pictures, we may still take advantage of the low-level features learned in ImageNet. A less aggressive approach may train the full network on an easier problem (such as data with more or less resolution) and then retrain the same network on a more difficult problem starting from the already learned coefficients.
- Ensemble networks and metaheuristics: Accounting for the possibility of getting trapped into local minima and not finding suitable parameters that perform a useful regression, many solutions include training multiple networks and combining their predictions in some way. This approach is known as an ensemble network. Related to this approach is the combination of known global optimizers such as genetic algorithms, swarm, or stochastic optimizers with the local optimizers typically used in neural networks. In general, these approaches are called metaheuristic optimizers.
- Hyperparameter optimization: Another approach that has been found useful is to optimize the network with respect to its architecture using cross-validation, i.e. the use of two independent sets of data to validate the weights of a trained neural network. For instance, we may change the number of hidden layers, their activation function, the number of neurons, or any other relevant aspect of the network.
- Dropout: The number of parameters of a neural network may be rather large, and it is possible that they may adapt very well to the training data but that they fail when trying to work on images that have not been seen during the training (even if they have the same aspect). One of the main techniques to avoid this overfitting is called dropout, and it consists in setting a random subset of the outputs of a layer to zero. This random subset is different at every batch of training data. In this way, the network is forced to introduce redundancy in its internal representation as it never knows which neurons will be dropped out at each iteration. When the network is used in reality, the dropout is not active, and the information flows redundantly throughout the network.
- Multiobjective learning: It has been experimentally observed that networks that have to simultaneously learn two or more objective functions, that is tasks like classifying images into distinct classes and segmenting the foreground object from its background, tend to learn better and be more generalizable to other inputs not seen during the training phase.

**Figure 6:**
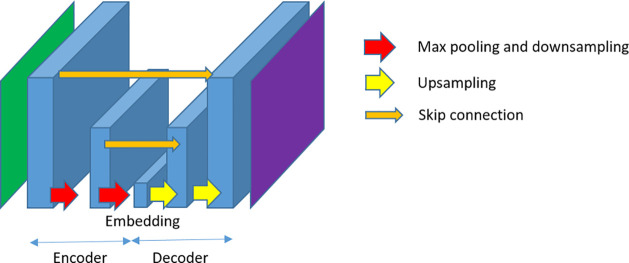
Example of Unet architecture. This is a complex network composed of different layers and connections, but it follows the architecture encoder-decoder.

**Figure 7:**
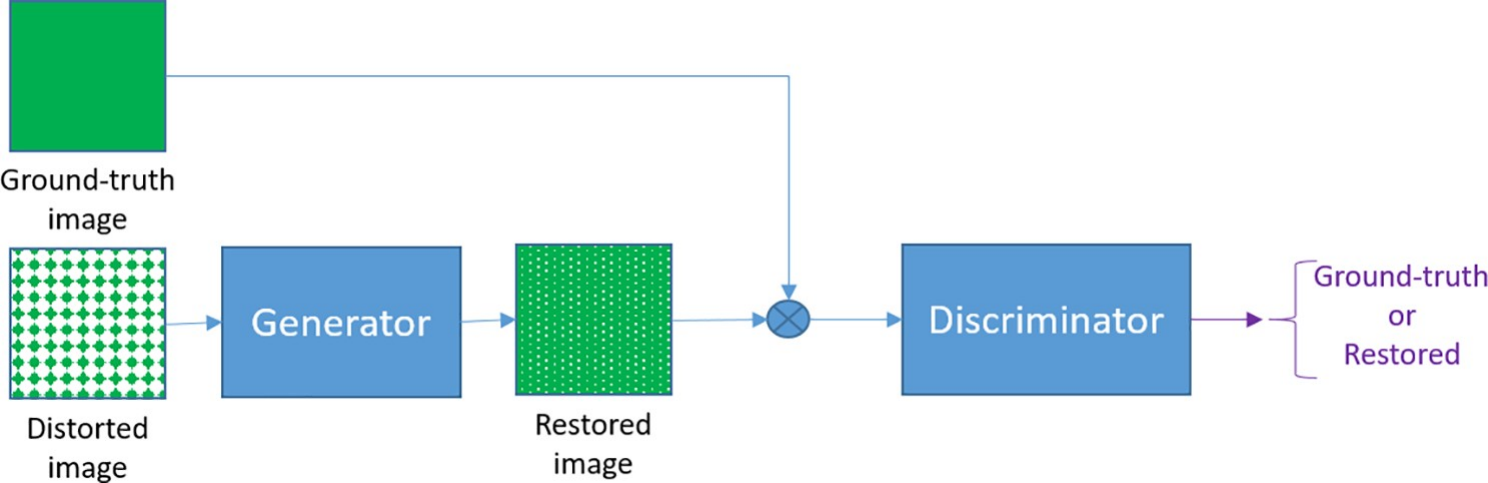
Example of the GAN architecture. The generator attempts to produce images as similar as it is possible to the ground truth. A second network chooses between the restored image and the ground truth.

The possibilities for combining neurons in a full network can be infinite. In general, these combinations are called the network architecture. We have already seen the dense (Figure 3) and the convolutional architectures (Figure 4). Besides these two, the architectures that have had the most impact in CryoEM are as follows:

- UNet: This architecture combines convolutional, downsampling, upsampling, and skip connections to produce a small representation of the information content of the input image into an embedding vector that later on is expanded into an output that is another image of the same size as the input (Figure 6). The goal may be (1) to produce an output image that is as similar to the input as possible, as is done in denoising, and the whole network is said to be an autoencoder (in this case, we would not use skip connections), or (2) to produce an image that is related to the input image, as is the case of segmentation or object location in particle picking. The number of hidden layers, their size, the number of kernels in each one of the layers, etc. may change from one implementation to another, but the overall idea remains similar.
- Variational autoencoders (VAEs): the architecture and goal of these networks are similar to those of the autoencoders presented above, but instead of predicting an embedding vector that is later decoded into an image, they produce a mean vector and variance. Then a random vector is drawn from a fixed distribution, typically a multivariate Gaussian with these parameters, obtaining the vector that must be decoded. The random draw of the embedding makes the network generalize better to unseen data.
- Generative adversarial networks (GANs): This architecture can be used for several purposes; here, it is shown with an example of image restoration (for instance, denoising) for a simple illustration (see Figure 7). Two networks are trained simultaneously: a generator and a discriminator network. Given a degraded input image, the goal of the generator network is to produce an image that resembles the ground-truth image as much as possible. A switch randomly chooses between the ground-truth and the restored images. The discriminator network must determine whether the presented image is really a ground-truth image or, on the contrary, an image produced by the generator. The loss function is such that the generator network tries to fool the discriminator, minimizing its classification success. Once both networks are trained, the generator network is used independently to restore degraded images. An interesting property of GANs is that the generator learns the statistical distribution of the input domain. There are many variants in which, for example, we may add the similarity between the restored and the ground-truth images to the loss function.

## 3 Classical Pipeline of SPA: An Overview and Methods

The capacity to structurally solve purified macromolecular complexes by CryoEM is due to the convergence of advances in sample purification, electron optics, image acquisition, and image processing. Once the sample has been purified, grids are produced and introduced into the microscope, and the images are acquired. A set of image processing techniques are used to determine the structure of the macromolecule under study. This set of techniques are collectively referred to as single particle analysis (SPA).

The starting point in obtaining the 3D structure of a macromolecule will be the set of images acquired by the microscope. Using these images as input, the SPA workflow is based on two hypotheses:

1. *Identical copies assumption*: all images of the macromolecule on the micrograph are images of multiple copies of an ideal canonical complex in the same conformational state.
2. *Projection assumption*: all acquired images are projections of the canonical complex under different directions.

Assumption 1 is a strong condition since macromolecular flexibility is very much linked to function. Indeed, most macromolecules present some degree of either flexibility or compositional heterogeneity. Therefore, this identity assumption will often result in a first approximation of the specimen structure at low resolution. The search for solutions to this heterogeneity problem is a current trend in methods development; this problem will be explained in-depth in section 10.

Assumption 2 is weaker; it considers that the sample is so thin that it interacts with an electron only once and that all planes of the sample (perpendicular to the beam axis) will be focused on the same plane with the same magnification (this is not strictly true because of the Ewald sphere, but this is a relatively minor refinement). Summarizing, the first assumption imposes a specimen condition while the second imposes an imaging formation condition. However, two extra considerations have to be made regarding the image formation:

- The information collected by the electron microscope is delocalized into a region related to the point-spread function (PSF) in a linear approximation of the microscope as an optical system. This delocalization makes it so that the collected images do not behave as pure mathematical projections of the 3D object under study but that they are further blurred by a PSF. The delocalization depends on the image acquisition defocus and spatial frequency (resolution).^48^ The Fourier transform of the PSF is known as the CTF (contrast transfer function).
- The interaction of electrons with the sample makes the latter move under the action of the electron beam. This is known as the beam-induced movement (BIM). The introduction of direct electron detectors allows acquisition of images in a very short time (on the order of a few milliseconds). Each of these images is referred to as a frame, and typically several tens of them are recorded. During these short periods of time, we may assume that the BIM is small. However, frames have to be correctly aligned to each other to recover the structural information on each macromolecule; then, they are summed up into an image referred to as a *micrograph.* If frames are not aligned, the misalignment results in a blurred projection due to the macromolecules’ motion.

CryoEM images are highly affected by noise. Between 10 and 100 times more noise power than signal power (SNR = 0.1–0.01) is present in the micrographs, and 1 order of magnitude less, at the level of frames. This represents a real challenge: image processing algorithms must be robust enough to deal with such an amount of noise while avoiding overfitting, local minima, or artifacts. The origin of the noise has several sources. At the level of frames, the noise follows a Poisson distribution (shot noise). However, at the doses normally used in CryoEM and after aligning and averaging the frames, the noise is normally distributed, and the most important source of noise (in the sense of the interfering signal) at that level is the amorphous ice layer surrounding the specimen.

Because the ice is amorphous and independent of each embedded particle when particle images are averaged, and due to the central limit theorem, the resulting noise of the reconstructed map is Gaussian.^171^ A second, but much less important, source of noise is the random arrival of electrons to each pixel. Due to the low total dose regime, between 30 and 60 e/Å^2^, the intensity distribution at each pixel follows a Poisson distribution with a mean that depends on the dose and the pixel size.

**Figure 8:**
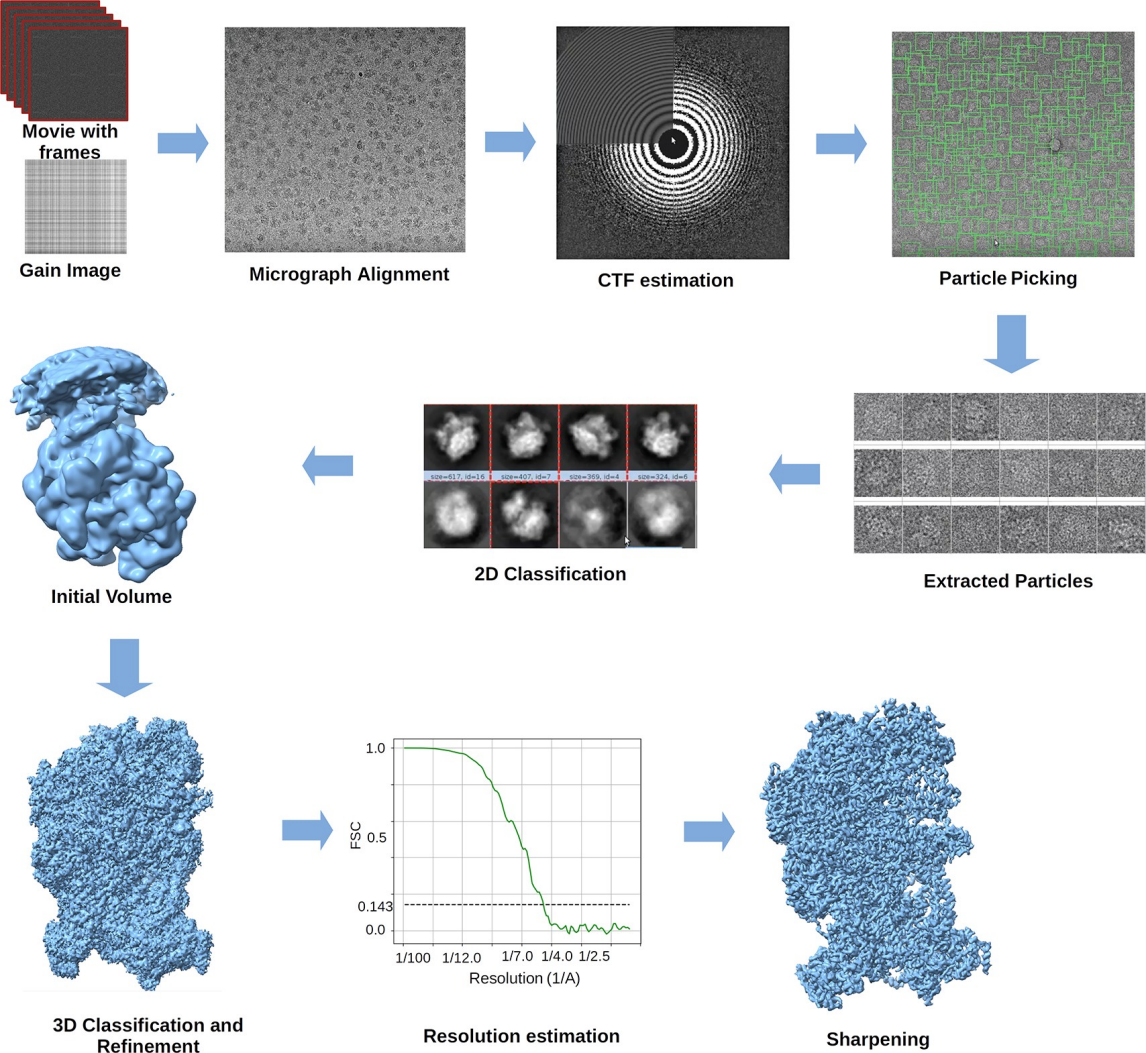
SPA workflow. The images are acquired as movies (frame collection). They are aligned to correct the beam-induced motion and averaged to reduce the noise variance. Then, the CTF is estimated to correct the microscope aberrations and defocus. Particles are selected to be later classified and screened in a 3D classification used to refine the structure. Finally, the map is sharpened to enhance the visualization, helping to build the atomic model (if it is possible).

The SPA image processing workflow refers to a set of image processing steps that allow for reconstructing the underlying 3D distribution of the electrostatic potential of the macromolecule from the set of 2D images acquired by the electron microscope. In other words, the workflow is all about determining the unknown projection direction of the imaged projections, called *particles*, and inverting the projection process. This is a hard task at the low SNR that the images present. Therefore, determining the 3D structure requires solving a large set of smaller but nontrivial problems to ensure the reliability of the reconstructions. These problems are the different steps of the SPA workflow. In Figure 8, we show the general set of steps. Experimental workflows may be much more complicated than the one shown here, but this simplification allows us to grasp the main steps along the path. As Figure 8 shows, the workflow starts with the *movie alignment*, where the motion of the particles as a consequence of the beam is corrected. The result is a set of images with the motion-corrected named *micrographs*. Next, we estimate the PSF, or its equivalent in Fourier space, the *contrast transfer function (CTF)*. Then, the *picking* step identifies the particles in the micrographs, distinguishing them from their ice surrounding and trying not to be fooled by contaminants, aggregation, crystals, carbon edges, or any other undesired signal. Particles are grouped according to their similarity through the *2D classification*. The *initial volume* step provides a first estimate of the structure. The initial volume is enhanced during the *refinement* and *3D classification* steps, and possible conformations are elucidated. Finally, the *map quality (resolution analysis)* is measured, and a *sharpening* is applied for enhanced visualization.

In the following sections, the SPA workflow is explained step by step: first, with a general overview of the problem that each step aims to solve and showing the main issues arising in its quest; second, showing the image processing methods (with a brief explanation of some of them) that have been developed in the last 10 years. Because one of the goals of this article is to review the use of analytical approaches and deep learning methods, the distinction between both groups of methods will be highlighted. Finally, at the end of each step, we will show a figure with the time evolution of the number of related publications with that workflow step. Our objective is to identify trends in methods and understand the algorithms’ working mechanisms rather than provide an exact number of publications for each year.

## 4 Movie Alignment

### 4.1 The Movie Alignment Problem

As we already mentioned, illuminating with electrons induces a movement in the sample due to the interaction of electrons with the matter. This displacement can be local or global. The movie alignment step is responsible for correcting these errors between frames, generating the so-called micrographs as the sum of the motion-corrected frames. The electron dose received by the sample accumulates during acquisition: the first frame is scarcely radiated, and the last one is highly radiated and, therefore, can be substantially degraded. To alleviate this problem, it is common to apply a dose weighting schema to the frames after the movie alignment. The effects of radiation on the sample have been previously analyzed in the literature,^45,46,58^ and certainly, they represent a limiting factor in the quality reconstruction.

Another common correction in this step is the gain correction of the camera, resulting from the fact that the sensitivities of all the sensor’s pixels can be different. They depend on the manufacturing of the camera and their current internal currents and potentials at the moment of acquisition as a semiconductor device. These differences evolve and have to be determined for each microscopy session.

### 4.2 Movie Alignment Methods

The first works on movie alignment attempted to characterize and correct the movement of the sample during acquisition as a consequence of radiation. From a pure image processing point of view, the need to address errors due to movie alignment was highlighted in the work of Brilot et al. in 2012,^19^ where the cross-correlation between images was used to determine the shifts between frames and correct for them. This simple approach drew some important conclusions: (a) Particle motion presents correlation in a radius of 300–500 nm. (b) Low electron doses reduce the particle movement. (c) High-resolution features can be recovered by aligning the frames. This work showed the need for movie alignment algorithms to achieve high-resolution reconstructions. As a consequence, during the following years movie alignment became a hot topic. Perhaps the most famous breakthrough was the development of MotionCorr^88^ in 2013. MotionCorr aligns the frames by introducing constraints in the frame motion. Essentially, it considers that the movement between any two frames (for instance, frame 1 and frame 4) must be the vector sum of the displacement vectors of all in-between frames considering the relative displacement between them (**r**_14_ = **r**_12_ + **r**_23_ + **r**_34_). The vector sum constraint avoids the situation where the occasional spurious correlation peak produces a large error in alignment. MotionCorr considers whole frame displacements. The advantage of this algorithm was its computational efficiency due to its GPU implementation and the reliability of the obtained results. MotionCorr was almost simultaneous with several other algorithms such as those introduced by Shigematsu and Sigworth,^157^ also addressing issues of pixel noise associated with dose, and Bai et al.,^8^ where they considered that particle displacements should be small and proposed the use of a Gaussian prior to determine them. This latter work was extended by Scheres^144^ adding spatial correlation in the movement of the particles, assuming close particles should present a similar behavior and introducing dose weighting. They did so by minimizing a merit function that takes into account the spatial correlation, using a Gaussian regularizer that depended on the distance between particles. The result of the minimization is the shifts and in-plane rotations of the particles. In 2015 two new algorithms were published. The first one addressed the problem of local motion by defining an objective function and optimizing it; to do that, the first derivatives of the cost function were analytically obtained by Rubinstein.^135^ The merit function considered the correlations of the Fourier transforms of each frame with the sum of the shifted Fourier transforms of the frames. The second algorithm introduced a novel approach, the use of the *optical flow algorithm* to estimate the local motion of the particles.^3^ Other methods, such as Unblur, address the alignment by introducing the electron dose and its effect on the SNR^50^ as weights. In 2017, the algorithm of MotionCor was improved, and *MotionCor2* was released.^228^ It introduced anisotropic correction of the BIM and described the sample deformation through a polynomial fitting. Thus, it can correct local deformations of the ice and local motions. This correction is carried out in two steps: first, a whole frame motion and, later, the correction of the anisotropic local motion. Also, in 2017 the package Zorro was released.^99^ Zorro performs the drift correction by cross-correlation using a noise model to weight each cross-correlation and filter. Finally, FlexAlign^183^ was developed in 2020. FlexAlign can carry out motion correction in real time thanks to its implementation in GPUs. Hence, it performs the movie alignment with a global correction similar to MotionCor, using B-splines and control points for local correction.

Movie alignment is the first step of the SPA workflow. The field is evolving toward image processing in streaming (as soon as the microscopes acquire the images, they enter into the SPA image processing workflow) and automation. Despite all current movie alignment methods being well automated, the new generation of detectors presents a greater number of pixels and the acquisition speed is getting faster and faster. It is not surprising that improving the speed and computational efficiency of the algorithms is a trend in movie alignment.

**Figure 9:**
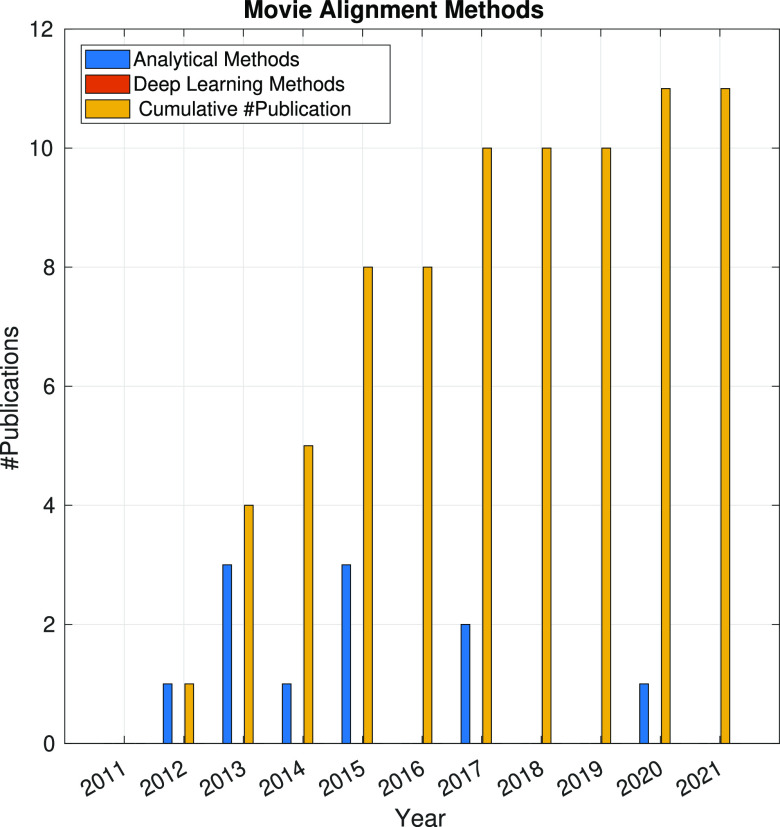
Time evolution of the number of publications about movie alignment based on analytical approaches or deep learning methods. The symbol #publications denotes the number of publications.

In Figure 9, the time evolution of the number of methods addressing the BIM correction is represented. Note that, at this stage, all algorithms are analytical approaches. Also, it is observed that movie alignment became a problem of interest in the years 2013–2015, with the interest in new methods declining since then.

It is of utmost importance to end this section with a remark that the best BIM correction would be to avoid the movement to start with. Indeed, this has been a topic of research in the past decade,^106,137^ directly related to a deeper understanding of the physical processes behind these movements.^111,138,140^ Recent works in the area^105^ may indicate that a significant physical movement reduction can be obtained by better understanding the process and a new EM grid design.

## 5 Contrast Transfer Function Estimation

### 5.1 The CTF Estimation Problem

The acquired micrographs are affected by the defocus and aberrations of the electron microscope. Although defocus is usually considered an aberration, it is not strictly so because it is not an imperfection of the optical system; in CryoEM, defocus is intentionally introduced by the researcher to gain some contrast between the macromolecules and their background. Aberrations are caused by defects, misalignment, or imperfections of the microscope optic, resulting in blurred and distorted images. The contrast transfer function (CTF) is the Fourier transform of the point-spread function, and it models the microscope aberrations, including the defocus. Note that the higher the desired resolution, the more critical the CTF estimation and correction are. The nominal defocus is known during image acquisition as well as the theoretical magnitude of some of the microscope aberrations, such as the spherical aberration coefficient. However, these nominal values may not be accurate enough. Hence, estimating the defocus and aberrations from the acquired micrographs is necessary for their posterior correction.

CTF estimation is normally carried out by analyzing the micrographs’ power spectral density (PSD). These PSDs have distinctive rings called Thon rings caused by the defocus;^195^ note that the electron lenses make the different diffracted components in the focal plane converge; however, due to the introduced defocus and the rotational symmetry of the lenses, defocus appears as rings (Thon rings) of spectral information. The amplitude of the Thon rings helps to determine other microscope aberrations. Traditionally CTF estimation algorithms work by fitting the power spectrum with |CTF|^2^ to estimate the defocus, astigmatism, or phase shift. This approach is justified since a random image, filtered by the CTF, will have a power spectrum that goes with |CTF|^2^. Currently, higher-order aberrations, such as beam tilt, are also estimated, although these are not estimated on the PSD but on the Fourier coefficients of the acquired images.

Traditionally the CTF estimation is initially carried out for each micrograph. However, particles can be at different heights inside the ice layer, and as a consequence, each particle has its own defocus. In the early stages of the SPA workflow, the CTF correction neglects this effect, which is normally corrected at the end of the workflow to refine the obtained structure.

### 5.2 CTF Estimation Methods

There are many methods for estimating the CTF, but they can be classified into three groups: estimation of untilted samples, local estimations of the CTF-per-particles, and estimation of tilted samples.

In the first group, we find one of the most used methods, CTFFIND,^101^ that carries out a fitting of the model of the microscope (the CTF) to the PSD of the images. In its latest version, CTFFIND4,^132^ the method was enhanced to include the effect of the dose and the use of phase plates (see below), and its performance was boosted. Another popular algorithm is gCTF,^225^ which provides a fast estimation of the CTF thanks to its implementation in GPUs. The CTF estimation is determined by maximizing the correlation of the CTF model with the difference between the PSD and the background. Another method is FASTDEF,^202^ an automatic and fast estimation of the defocus that does not require an initial defocus for the estimation. FASTDEF uses a Zernike polynomial basis to estimate the aberrations. Whenever a physical magnitude is measured, its associated error should also be estimated; aberrations and defocus are not an exception. Thus,^112^ they proposed CTER, an efficient and accurate algorithm for the CTF and its uncertainty estimation. It is very common that two different algorithms estimate different parameters for the same micrograph. In the community effort referred to as the CTF Challenge,^95^ it was reported that the typical uncertainty of the defocus of a micrograph was between 200 and 300 Å, although we should note that this accuracy has undoubtedly improved since then judging by the field capacity of obtaining maps below 2 Å resolution. Sheth et al.^156^ proposed a way to measure the consistency between the estimated and the observed PSDs to define the resolution of a micrograph. Furthermore, multitapering was recently proposed by Heimowitz et al.^57^ to reduce the bias in the estimation of the PSD by applying multiple Slepian functions as mask windows.

The second group considers methods that provide an estimation of the CTF per particle. gCTF^225^ also allows this estimation. To do that, gCTF makes use of the neighbor pixels around the particle and then considers an initial estimation of the global CTF of the micrograph, using it to refine the local estimation per particle. Recently, Zivanov et al. proposed a method to estimate higher-order aberrations such as tilt, comma, or trefoil, by combining reprojections of the map and the use of a Zernike decomposition of the CTF argument.^234,235^ This method requires a high-resolution reconstruction of the macromolecule, and for that reason, it is explained in more detail in section 11.

The third group considers the CTF estimation of tilted samples. It was recently pointed out that tilting the sample in the microscope can increase the angular coverage of an acquisition.^189^ This is particularly useful if there are preferential interactions of the protein with the water–air interface. goCTF^184^ was specifically designed to estimate the CTF in such tilted samples. The dependence of the defocus on the height has been long known for large specimens, and several methods have been proposed to correct this dependency within a single particle.^97,211,212^ The implications for tomography of these developments are obvious, but electron tomography methods are beyond the scope of this review.

Finally, we would like to comment on the use of phase plates in CryoEM. These devices allow for working in focus without losing contrast and have reached significant technical development with the so-called Volta phase plates (VPPs).^27^ Assuming that the microscope is aberration-free, to work in focus allows one to neglect the CTF correction. However, working in focus also makes the Thon rings disappear and complicates the CTF estimation, which is crucial since some kind of aberration is always present.^28^ More developments in this area are expected in the coming years.

**Figure 10:**
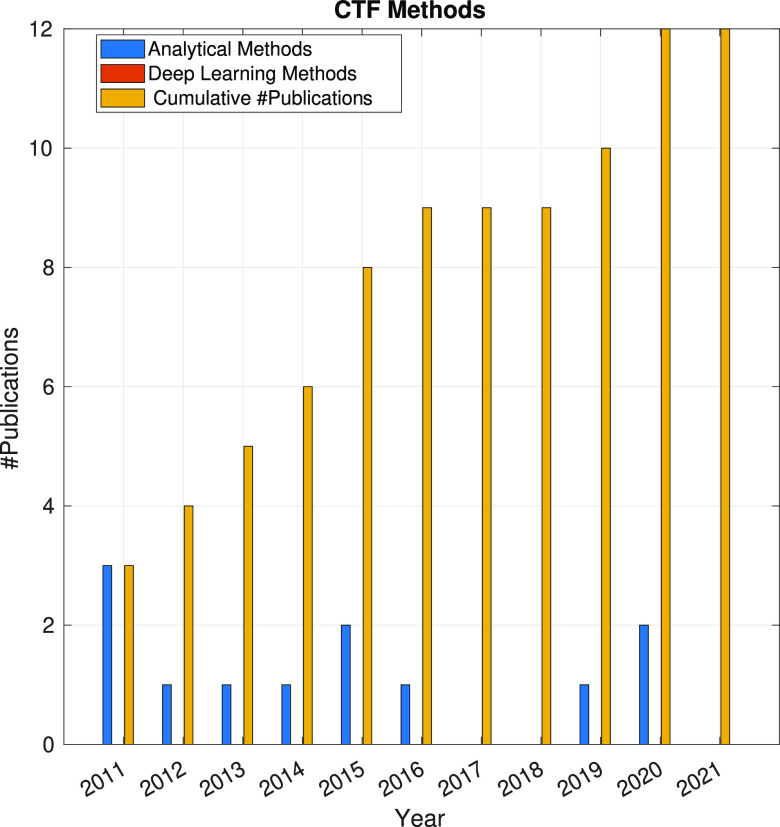
Time evolution of the number of publications of CTF estimation based on analytical approaches or deep learning methods. The symbol #publications denotes the number of publications.

The trend in the publication of CTF-related methods shows a constant publication rate (see Figure 10), first, with the publication of methods for untilted samples and, now, with the new methods on local CTFs. In our opinion, the current trend points toward faster estimations, with improved accuracy (reliability) and the estimation of high-order aberrations. The use of tilted samples also seems a new topic, but it complicates the image acquisition and the CTF estimation.

## 6 Particle Picking

### 6.1 The Particle Picking Problem

Each micrograph contains projections of many copies of the structure under study; these projections are called *particles*. Particles are selected and cropped from the image to input subsequent image processing steps in the picking step. The atomic number of the elements that compose the specimen is very close to the atomic number of the aqueous solution where the macromolecules are. As a consequence, the contrast of the particles in the micrograph is very low. The contrast can be increased by defocusing the sample, but then the high-frequency information content of the macromolecule is compromised. The picking step is critical in the SPA workflow, since the reliability of the reconstructed structure will depend on the quality of the selected particles. Particle picking algorithms work by searching micrograph areas with specific features similar to those of the particles sought after. However, other regions in the micrograph may spuriously correlate with the kind of object we look for, resulting in false positives. A data set with a significant amount of false positives is rather dangerous because of the so-called *Einstein-from-noise* effect^59,198^ (the average of noise particles aligned to a reference looks like the reference) and represents a threat in the SPA workflow due to the possibility to produce wrong but very reproducible structures as a consequence of the results being biased.^168^ A similar effect is observed if an incorrect macromolecular template is used for picking the particles.

### 6.2 Picking Methods

Leaving manual picking aside, picking methods are usually classified as semiautomatic or automatic, depending on the degree of interactivity required from the user. This review will show analytical and deep learning picking methods, and we will follow their yearly evolution in the past decade.

Starting with the group of analytical methods, we find Xmipp-picker,^4^ a semiautomatic picker. This picker is trained with a small set of particles that the user has to select manually (around 15 particles). From this set the picker learns how to distinguish particles and noise by means of a classifier based on support vector machines (SVMs) and a number of engineered image features. gEMicker^66^ uses normalized cross-correlation to find the particles in the micrographs using templates (class averages or specific particles as input) of the particles to be sought after. The novelty of this latter picker was its implementation in multiple GPUs, making it a very fast picking tool. Autopicker/ViCer^85^ finds particles following a two-step strategy: first, the Autopicker algorithm carries out template matching to select particle candidates; then, a refined set of candidates is obtained by means of principal component analysis of the obtained particles and the application of the Otsu algorithm;^110^ finally, the ViCer algorithm performs an outlier detection with an unsupervised classifier. Yet another approach to the picking step takes advantage of the existence of relevant similar structures obtained in many cases from X-rays. If they are available, they can be converted into density maps and projected to obtain a gallery of templates. Thus, Rickgauer et al. showed that by means of template matching it is possible not only to find the particles in the micrograph but also to determine their orientation.^131^ As will become clear in this section, variations of template matching are highly used for analytical picking methods, with many other proposals such as the ones used by RELION^78,146^ or Gautomatch,^1^ which uses GPU support to increase the performance. Additionally, template matching is also closely related to other fully automatic approaches that do not explicitly use templates as input (i.e., the user is not asked to provide templates), such as APPLE picker,^56^ where the templates are internally estimated. Finally, pickers specifically designed for helical particles also exist, such as in ref (68), but are not covered here.

In the group of deep learning pickers, the algorithms use neural networks to undertake the picking. These networks are previously trained with CryoEM images to make a fully automatic picking possible. Also, many deep learning methods allow training and/or refining the model with the data set at hand. In this class, we find DeepPicker,^215^ which performs the picking by means of the first step of a convolutional neural network (CNN) designed to find candidates to be particles; this is followed by a second layer which carries out a classification of whether the candidate is a particle or not. Topaz^15^ represents another picker also based on a CNN composed of eight layers that alternatively combines a convolutional layer with subsampling layers finishing in a fully connected layer. Topaz preprocesses the micrographs to find those regions with a high probability of containing particles. The search for these regions is defined as a positive and unlabeled learning problem. DeepCryoPicker trains the CNN with unsupervised learning algorithms and is designed to work with extremely low SNR.^5^ Warp also includes a picker based on BoxNet, a fully convolutional ResNet architecture composed of 72 layers.^190^ crYOLO^213^ makes use of the general YOLO network (you only look once)^130^ approach specialized to the CryoEM picking problem. YOLO consists of 22 convolutional and 5 max-pooling layers. To avoid the limitations of YOLO with small particles, micrographs are divided into a small number of overlapping patches. PARSED^222^ performs the picking from a segmentation point of view. To do that, particles are found by means of a fully convolutional neural network composed of only convolutional and deconvolutional network layers. Finally, PIXER^224^ uses a segmentation network to create probability maps (of finding a particle) from the micrographs.

Despite the many available automatic and semiautomatic methods, particle pickers end up selecting a non-negligible number of incorrect particles. Consequently, the task of particle quality assessment and sorting is important. Tools such as those introduced by Vargas et al.^199^ can be used to separate gross erroneously picked particles from correct ones based on multivariate statistical analysis of the particle set. MAPPOS^108^ is a pruning algorithm that uses a classifier to determine if the particles are correctly picked. To do that, the user has to train the classifier with a small subset of particles. Pruning methods in the field of deep learning also exist; one of them is deepConsensus.^142^ It receives the set of picked particles by different picking algorithms, and utilizing a CNN, the picked candidates are classified as true or false particles. The problem of discriminating between particles on carbon and particles on ice is also addressed by first detecting carbon supports using EMHP.^17^ A different approach is micrographCleaner,^143^ which is a segmentation tool based on a trained Unet-like model that excludes those particles that lay on undesired regions such as carbon areas, contaminated regions, or regions that are simply artifacts.

**Figure 11:**
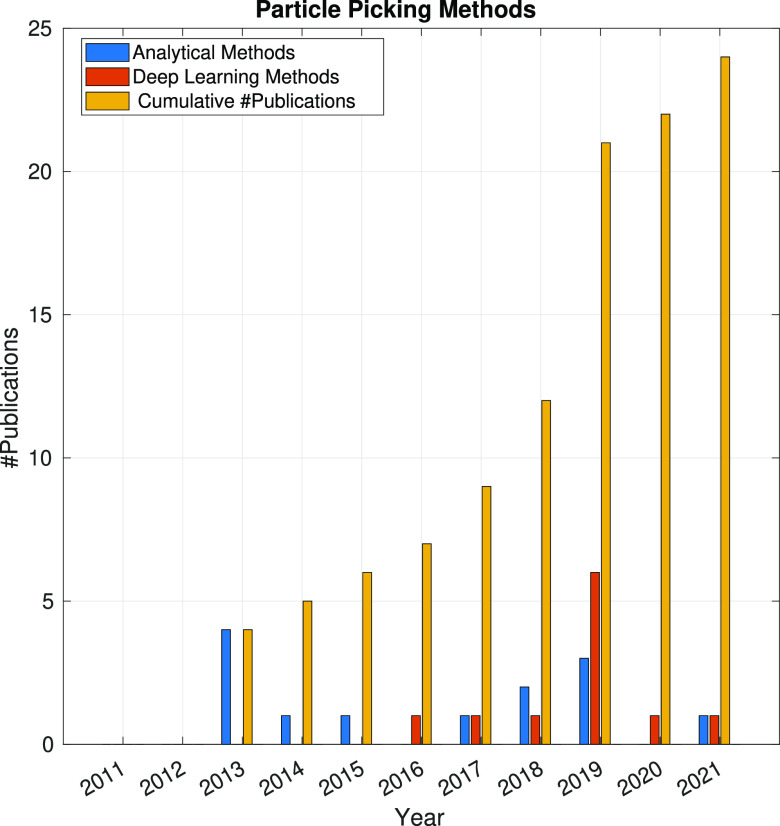
Time evolution of the number of publications of picking based on analytical approaches or deep learning methods. The symbol #publications denotes the number of publications

As can be easily inferred by the large numbers of picking algorithms developed over the years, this is not a generally solved issue in CryoEM. Figure 11 shows the number of new articles on this topic over the years. We highlight the increased interest in recent years. Probably the reason for this trend is the introduction of deep learning approaches, as Figure 11 suggests. Indeed, picking particles is a task that is very close to the standard formulation of deep learning algorithms and, consequently, has been one of the first ones to benefit from this new technique. Some of these algorithms have been trained on tens of previous projects to be applied to new projects without retraining. This possibility helps to automate the SPA image processing pipeline further. Other topics of interest are identifying nonparticles such as interfering areas of the carbon support film, ice contamination, or malformed macromolecules. Finally, we would like to highlight the benefits acquired from the existence of this wide variety of picking methods that allow for applying consensus techniques to further ensure the reliability of the results.

## 7 2D Classification

### 7.1 The 2D Classification Problem

Once particles have been selected, they are grouped by similarity into different sets called *2D classes*. Generally, similar images will have close projection directions. As particles come from the picking step, they can be in any arbitrary orientation with respect to their class representative and normally need to be aligned. Their relative shift must also be determined. We may think of the class representative as the weighted average of all the particles assigned to that cluster once aligned, which is much cleaner than the raw particles. Images are supposed to have been normalized in preprocessing steps so that the surrounding noise has zero mean. By averaging, the noise variance is reduced, and the signal of the particle is reinforced. In many cases, it is possible to visualize the projection of secondary structure elements, such as α-helices. These high-resolution features in the 2D classes are a good indicator that it might be possible to obtain a high-resolution reconstruction. In essence, the goal of the 2D Classification step is twofold:

1. As was pointed out previously, the low SNR in the image particles compromises the accuracy of the picking. Indeed, picking pure noise particles and artifact-like defects is relatively common, despite the continuous advances on pickers reviewed in the previous section. These undesirable particles affect the quality of the reconstruction. The 2D classification step helps to group these unwanted images. Undesirable classes will generally contain undesirable particles, but the opposite is not necessarily true (not all images assigned to a good class are good). The grouping into classes is not a warranty of obtaining clean sets of particles, and there always remain noisy particles and artifacts in many classes. This is the reason why it is usual to perform several rounds of 2D classification. In this sense, 2D classification is used as a screening step of the picked particle set.
2. Class representatives have a much higher SNR than raw particles, resulting in a lower uncertainty during the 3D angular assignment. This is particularly important during the construction of the initial volume (section 9).

### 7.2 2D Classification Methods

Most 2D classification methods work by generating a fixed number of classes. Then, particles are classified iteratively, assigning them to one set by comparing them with the class representative using a similarity metric. Once particles are assigned to a class, the reference is updated with the new particles. This general strategy is called multireference alignment and has a long tradition in the field;^197^ note that, generally, particles are also aligned against the reference before computing the similarity score. Indeed, 2D classification is a crucial step in the analysis and understanding of the data; it is, therefore, logical that there have been new methods proposed in the last 10 years. We start the analysis of these methods with an elegant approach proposed in ref (163) by Singer. The method assumes that all particles are centered, and it focuses on the in-plane rotations and clustering. It works by estimating the distance *d*_*ij*_ between all pairs of images and the optimal in-plane rotational angle θ_*ij*_ between pairs of images. From these distances, the method estimates a sparse Hermitian matrix. The nonzero components of the matrix represent the rotationally invariant distances, and the main eigenvector describes images with a close rotation angle. The ISAC (iterative stable alignment and clustering) algorithm^221^ proposed a different solution. ISAC attempts to align and classify particles into highly homogeneous and stable classes, which is not an easy task due to their attraction problem—when highly populated classes exist, the lack of noise lowers the barrier to be assigned to that class, and many particles may be wrongly attracted to those highly populated classes. To minimize the attraction problem, ISAC automatically splits a class if its population is large enough (this strategy was already introduced in ref (166)). This is the essence of the EQK-means algorithm (equal-size group k-means). Another classification method addressed the use of robust w-estimators for class means.^67^ This w-estimators approach considers that particles are aligned against the reference, and images are corrected by the CTF. The method estimates the class mean as the fixed point of the weighted average of the images of each class. The weights are the product of two terms: the first one is the absolute value of the correlation coefficient between each image and the class average, while the second one, an exponential term, is responsible for limiting the number of outliers of each class. In terms of popularity, methods such as the expectation-maximization algorithm of RELION are prevalent due to their computational efficiency and GPU implementation.^78^

Common to all methods above is the use of local optimizers with a non-negligible chance of getting trapped into local minima. Probably the first method in CryoEM to explicitly address the need to increase the radius of convergence of the algorithms by introducing some element of randomness in the classification was PRIME-CLUSTER.^129^ Indeed, this algorithm introduced stochastic hill climbing (SHC) and random walk approaches in class estimation. The algorithm is similar to k-means, but it significantly differs in the matching process between particles and class averages. Traditionally, the identification of the best in-plane rotation angle was performed by maximizing the correlation. Instead, PRIME considers the first in-plane random rotation and cluster that improve the previous correlation. This simple random search reduces the computational time and alleviates the dependency on the classes’ initialization. Important elements of randomness in the optimization were later on introduced in RELION^235^ and CryoSPARC,^119^ especially through approaches such as stochastic gradient descent. Still, the field has been rich in proposals, such as NCEM,^160^ based on graph theory and using correntropy as the similarity measure. The use of statistical manifold learning was also proposed to solve 2D classification problems. The idea proposed by Wu et al. was to establish a correspondence between the input particles and a set of variables in a latent space by means of a generative topographic mapping.^217^

We note that most algorithms search for homogeneous classes under the hypothesis that all particles in the same class are rotated and shifted versions of the same projection. However, the reality is different. The starting particle set may be more heterogeneous than what was algorithmically modeled, with contaminating particles, artifacts, and false particles that are just pure noise or wrongly picked (besides aspects of macromolecular flexibility or compositional heterogeneity). Thus, there is still a need for methods to further screen and rank particles and classes, such as the outliers’ removal methods proposed in refs (18 and 177) or the automated approaches to detect good classes based on deep learning in refs (89 and 181).

**Figure 12:**
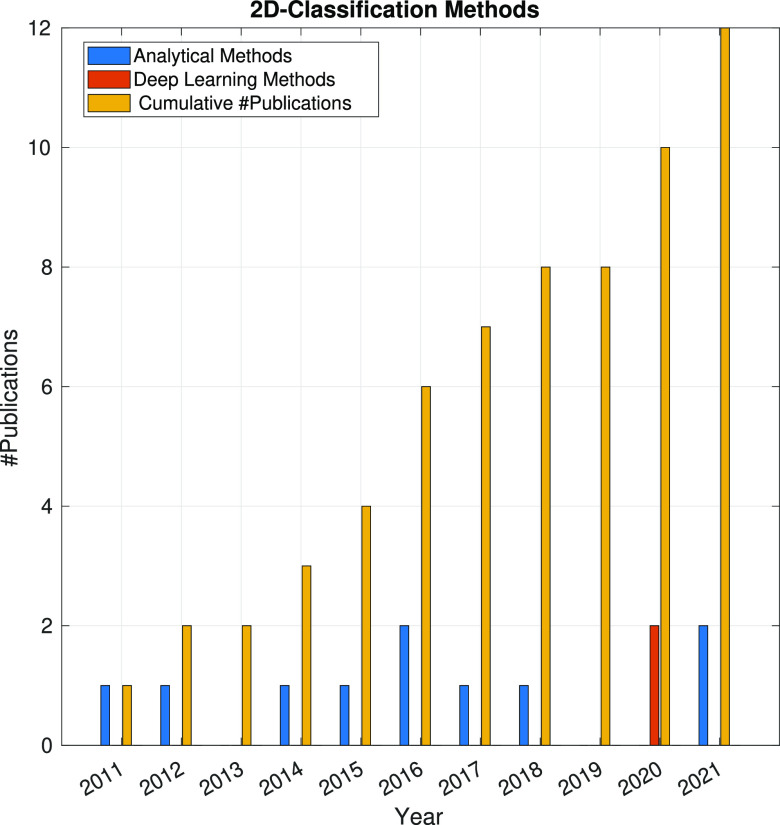
Time evolution of the number of publications on 2D classification based on analytical approaches or deep learning methods. The symbol #publications denotes the number of publications

As shown in Figure 12, most 2D classification algorithms developed in the past decade belong to analytical methods. This is not surprising because unsupervised deep learning algorithms are much less common than supervised ones. However, we expect more deep learning works to appear in the coming years. In fact, deep learning is already being used for 3D continuous flexibility analysis in an exploratory manner—as will be covered in other sections—so the algorithmic bases are there. In short, 2D classification methods have been focused until now on increasing the speed of the process, the use of different similarity metrics, and the reduction of the attraction problem among classes.

## 8 Reconstruction

This section is slightly different from the rest because it does not cover a specific step of the SPA workflow. In turn, reconstruction methods are so fundamental that they are applied in several workflow steps, such as initial volume, 3D classification, and map refinement.

The general problem addressed by 3D reconstruction algorithms can be stated in the following manner: we want to find the 3D structure from a set of its 2D projections under different and known points of view (projection directions). It is assumed that all projections have the same magnification. This problem is called tomographic reconstruction. Many methods have been designed to solve this problem; we mention some traditional approaches such as Fourier reconstruction^26,30,98^ and Fourier gridding,^113^ weighted backprojection,^121^ iterative algebraic reconstruction techniques such as SIRT or ART,^44,49^ and variants of these methods.^6,96^ This wide variety and the constant publication of reconstruction methods throughout the years highlight reconstruction techniques’ relevance. In SPA, Fourier gridding is the most used method due to its computational speed.

In the last ten years, we also find many publications on specific 3D reconstruction techniques for CryoEM. In this way, Li et al.^87^ presented a 3D reconstruction algorithm based on B-spline functions modeling the structure in 3D. The optimization was carried out by the *L*^2^-gradient flow of energy model solving a variational model with a TV regularization term, that is minimizing an energy term that combines fidelity to the experimental data and a penalization for rapidly varying maps. A later extension of this work by Xu et al.^219^ addressed issues related to its computational efficiency and accuracy. Along those lines, Kucukelbir et al.^81^ tried to find an adaptive basis of functions based on the data at hand to improve the map’s SNR. To select the adaptive basis, a Bayesian approach was considered by Wainwright under a sparsity prior.^214^ They observed that an appropriate frame for SPA reconstructions seemed to be stationary scaling functions and wavelets. The results seem to keep high-resolution information and to suppress background noise.

One of the problems of reconstruction algorithms is their high computational time. Trying to overcome this problem, the subspaceEM method was proposed.^37^ The algorithm required a set of aligned particles and an initial volume. The key idea was to eliminate all redundant information from the particles in the initial volume projections by performing a PCA (principal component analysis). Particles were then represented in a low-dimensional subspace where it is possible to rotate, align, and carry out transformations quickly, accelerating the expectation-maximization algorithm to reconstruct the structure. Abrishami et al.^3^ refined the gridding based direct Fourier method to explicitly consider the interpolation function used to map the Fourier coefficients of the images onto the Fourier coefficient of the volume. Without this correction, the results were as if the reconstructed volume would have been multiplied by a mask that had not been made explicit before. Thus far, the field of deep learning has only provided one reconstruction algorithm.^51^ The method named CryoGAN uses unsupervised deep adversarial learning to learn and identify the different poses of the particles.

An important consideration for reconstruction approaches is that, for thick samples, the acquired images are not pure projections of the samples due to the depth of field. Therefore, the Fourier transform of the particles will not be planes in the Fourier space of the reconstruction. This is the essence of the so-called Ewald sphere correction^139^ that should be applied when dealing with thick samples. Following this work, we expect that in the future more 3D reconstruction algorithms will explicitly incorporate more physical models of the image formation process.

**Figure 13:**
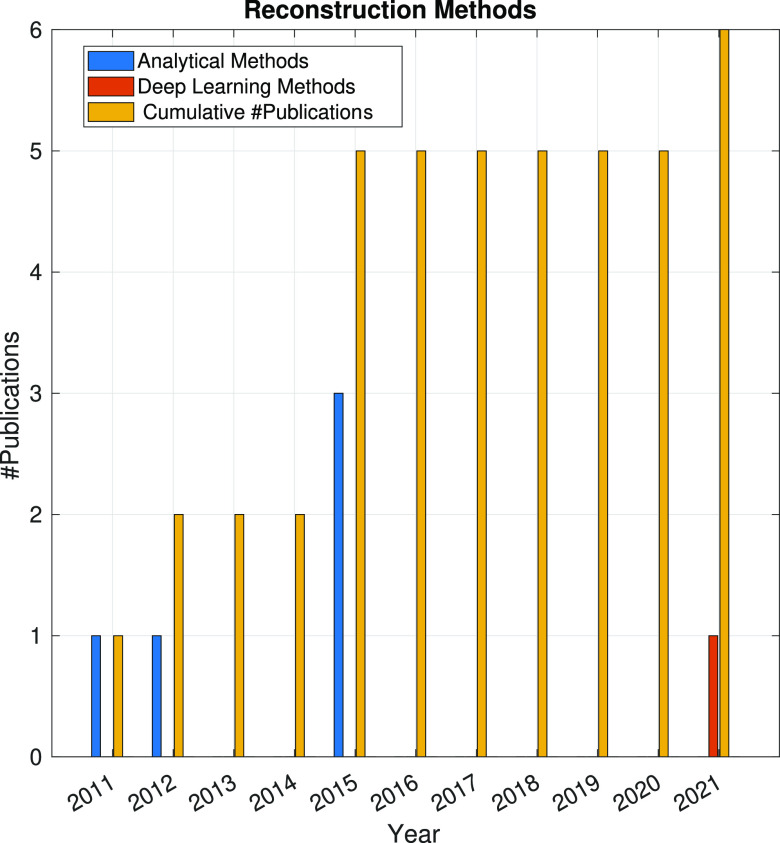
Time evolution of the number of publications on reconstruction based on analytical approaches or deep learning methods. The symbol #publications denotes the number of publications.

Most of the methods in this section belong to the category of analytical methods (Figure 13). Probably, the CryoEM community will be witness of the development of new reconstruction algorithms based on deep learning in the near future. The pros of analytical reconstruction methods are the combination of good computational performance, good quality of reconstruction, their capacity for dealing with low SNR, and the fact that they provide a result with clearly known mathematical properties.

## 9 Initial Volume

### 9.1 The Initial Volume Problem

We refer to this step as the provision of a first initial map (or maps) for the subsequent steps of 3D classification and refinement. Initial volumes do not need to be detailed reconstructions, and therefore, their resolution is normally low, some 20–30 Å. Despite their low resolution, the initial volume estimation is a critical point in the SPA workflow. Indeed, if this first approximation of the structure is wrong, then, in the most optimistic case, the convergence to the real structure will be slowed down. Still, in many other cases, an incorrect reconstruction will be achieved.

Algorithms to perform this step need to assign some approximate 3D angular orientation to subsets of particles or images derived from the particles. The former section shows that 2D classification reduces the complexity of many image processing operations by increasing the SNR. Disregarding the use of 2D class representatives or raw projections, initial volume algorithms must find a suitable volume that is compatible with the acquired data. However, the compatibility landscape has many local minima, and the two most powerful strategies employed to minimize their effect are (1) using a stochastic optimization algorithm and (2) smoothing the landscape. The past decade has been very active in trying to solve this problem, and we may subdivide these algorithms into three groups:

- Group 1. Algorithms exploiting the central slice theorem, which establishes that each particle (projection image) is a central plane of the Fourier transform of the reconstructed map. In this way, any pair of images should have a common line in Fourier space. Finding the common line gives information about the relative angular assignment between both images.
- Group 2. Algorithms that start with a random angular assignment. Despite their higher computational cost, they constitute the currently preferred family of methods due to their ability to find more representative initial volumes.
- Group 3. This group exploits the geometrical relationship between two acquisitions of the same area at different tilts. Although not much in use today due to the relative success of Group 2, this technique is handy for those cases in which purely computational tools fail.

### 9.2 Initial Volume Methods

In the first group of algorithms, we find a wide variety of proposals. In ref (158) the problem of finding common lines is posed as a synchronization problem; that is, the alignment parameters are estimated from relations between pairs of images. The solution is found by optimizing the number of pairs that keep a consistent relative spatial configuration. Wang et al.^236^ revisited this solution, and a more robust solution is proposed considering unsquared residuals and introducing a spectral norm term in order to avoid the clustering. The OptiMod method is also based on the search of common lines.^93^ Instead of reconstructing a single map, it generates multiple reconstructions, considering each one a rough solution. In a subsequent step by Pragier et al., they are all compared. Other initial volume methods also interpret the estimation of the initial volume as a synchronization problem.^116^

In the second group, we find PRIME,^38^ which employs an approach of random reconstruction and reprojection of the reconstruction to compare with the particles/classes. Each pair of experimental images and reprojections is given a weight relative to its correlation. Stochastic hill climbing is then used to accept or reject new orientation candidates. This algorithm was later improved by changing the stochastic optimization algorithm.^128^ In another work, the quite broadly used RANSAC (random sample consensus) algorithm was used for this task^200,39^ This method randomly assigns 3D orientations to a small subset of 2D class representatives and then evaluates the result with respect to the rest of the representatives. Those classes that correlate well with the reconstructed volume are called inliers. This process is repeated many times, and the volumes with the highest number of inliers are kept. Reconstruct significant^170^ is an iterative algorithm that computes the statistical significance for the similarity of each one for the possible class representative–reprojection pairs measured in multiple ways. The statistically significant pairs are then used for the reconstruction at the current iteration. The significance is progressively increased along iterations. A novel technique was introduced in CryoSPARC,^117^ which uses the stochastic average gradient descent (SAGD) in combination with the well-known maximum *a posteriori* estimation.

In addition, they introduced the importance sampling scheme, which is greatly responsible for the high computational speed of the method. In this way, instead of working with all possible rotations and shifts, it considers random subsets where the probability distribution of an image is optimized. Finally, Joubert et al.^74^ introduced a method that employs a pseudoatomic model with Gaussian functions. The model is combined with a Bayesian framework. All these methods may provide either a single initial volume or, instead, a set of candidates. However, practitioners may wonder if combining several initial volume estimates to achieve a consensus volume might still be more reliable or of higher quality than any of the candidates. This is exactly what swarm consensus^173^ does. It simultaneously uses the whole set of particles and a set of initial volumes estimated by different methods. Then, employing the swarm optimization, a stochastic gradient descend with momentum, the population of volumes evolves toward a more globally correct initial volume.

The third group of algorithms considers the classic technique of random conical tilt (RCT), which makes use of two images of the sample, one of them acquired with the sample tilted a given angle.^122^ The objective of this technique is to introduce the tilt angle as a constraint to simplify the search of the particle orientation, which is then performed just in two dimensions in the untilted images. In this group, we find ref (164), in which the theory behind random conical tilt was revised and generalized to situations in which the particles were not centered with respect to the reconstructed volume, as is normally the case due to the imprecision of the particle picking step.

**Figure 14:**
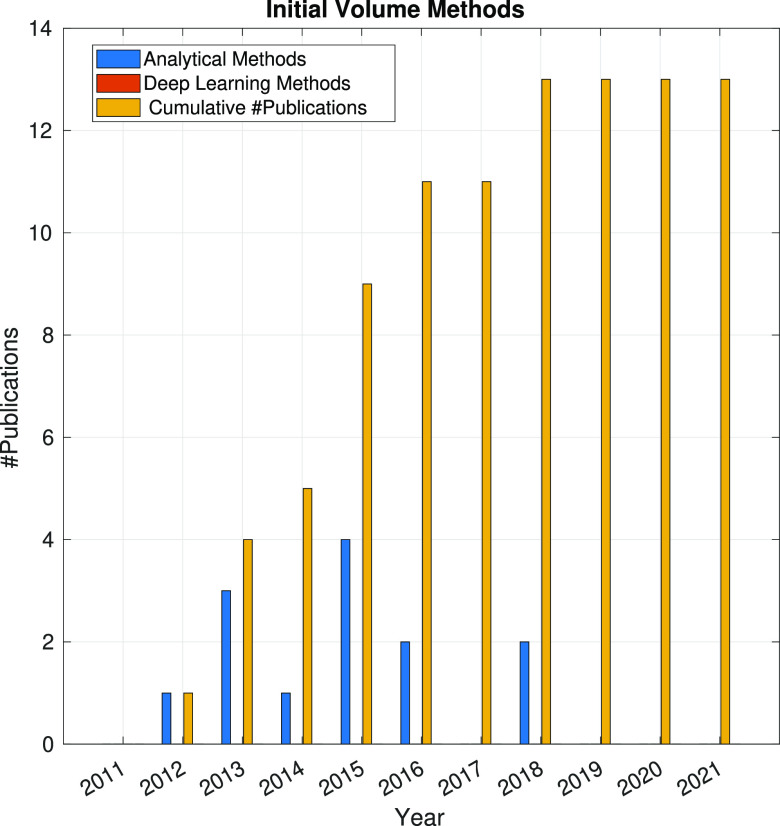
Time evolution of the number of publications on initial volume algorithms based on analytical approaches or deep learning methods. The symbol #publications denotes the number of publications.

We have shown in this section many methods for the initial volume estimation. All of them belong to the category of analytical methods. The estimation of a proper initial volume has been a constant subject of research in the past decade, although it seems that it has lost momentum in recent years (Figure 14). It is probable that we have currently reached a development plateau, which probably indicates that the quality of existing initial volume methods is already good enough for most purposes. However, the calculation of a representative set of initial volumes in the presence of large heterogeneity is still an open problem.

## 10 3D Classification

### 10.1 The 3D Classification Problem

Routine CryoEM grid preparation normally takes from seconds to minutes (with the freezing step itself being in the millisecond range), but this follows hours or days of biochemical manipulations to produce the sample. In this relatively large time, specimens are expected to sample most of the allowed conformational landscape under the experimentally set conditions of temperature and pH, among other factors. However, different conformations break the first SPA assumption, which considers that all particles are identical copies of the same reference macromolecule. This problem was previously referred to as *heterogeneity*. Its straightforward solution is to perform a discrete 3D classification by splitting the population of particles into subsets (classes) that attempt to capture the different states of the protein. Thus, each 3D class can be independently refined to reach a high resolution. This straightforward solution is currently the most used in the CryoEM field, although the situation is changing.

We can distinguish two kinds of heterogeneity: discrete and continuous. Heterogeneity will be discrete when we explicitly declare that the problem is splitting a data set of images into a finite number of relatively homogeneous subsets, with that number being an algorithmic parameter. In turn, we will refer to continuous heterogeneity when algorithms can deal with essentially continuous changes in the macromolecule without the need to set a defined number of classes to partition the data set; however, they may introduce other assumptions about how structural changes happen.

### 10.2 3D Classification Methods

Traditionally, 3D classification methods have focused on the search of discrete conformations, and in general, they need initial models of each of the conformations to be refined. One of the most used methods is 3D classification, as presented in the RELION package.^145^ It assumes that the number of structural classes is known, so particles are classified using a maximum *a posteriori* approach reconstructing the associated representative of each class. In turn, the software package FREALIGN^92^ carries out the 3D classification by expectation-maximization of a marginal likelihood, while the particle alignment is determined maximizing a joint likelihood including some hierarchical priors. A similar approach from a statistical point of view proposed by Zheng et al. suggests a mixture of Gaussians to represent each class; then expectation-maximization is used to estimate the parameter of the Gaussians.^229^ More recently, CryoSPARC^119^ made use of a branch-and-bound search strategy and a stochastic gradient descent (SGD) approach to perform *ab initio* structure determination and 3D classification, representing a new way to tackle 3D classification with a very high computational efficiency. In general, SGD is considered to be a robust method to search for deep optimum solutions in nonconvex problems like this one. Gupta et al.^52^ extended the GAN approach (see section 2.2) of ref (51) to consider the possible existence of multiple conformations.

Another distinct approach to address the heterogeneity problem is through the use of energy landscapes and manifolds. In general, macromolecules may be in different states, each one with a different free energy. The continuous motion of the macromolecule can be captured in a manifold in some abstract space (for a review on continuous heterogeneity, the reader is referred to ref (175)). This family of algorithms establishes a correspondence between the observed particles and their conformational state, that is a map between the particles and the conformational manifold.^43,153^ Currently, this approach is used by a series of different methods. The main difference between them is how to carry out the embedding into the manifold. For instance, Schwander^152^ suggests three different manifold embedding approaches: generative topographic mapping, Isomap, and diffusion maps. The use of energy landscapes was popularized with the work of Dashti et al.^29^ Particles were first aligned against a global reconstruction without taking into account their heterogeneity. Then, all particles with a close angular assignment were used to define a conformational manifold using a diffusion map embedding algorithm. Maji et al.^94^ explored how to “stitch” all the local manifolds calculated in ref (29). A different approach by Moscovich et al. and a more recent method constructs a manifold from a graph Laplacian defined from the projection images.^103^ A heuristic analysis of manifolds obtained with a simulated heterogeneous cryo-EM data set was used to build a framework from which reconstituting the quasi-continuum of conformational states.^154^ CryoDrgn^230^ proposed a method using a variational autoencoder architecture trained to encode the particle images in a latent space, the manifold. e2gmm^23^ is another deep learning based algorithm. Macromolecular flexibility is described by the different combinations of the parameters of a Gaussian mixture model (GMM). They proposed a neural network based on encoders to map the particles into a latent space and then decoded this latent space into a set of parameters for the GMM. Principal components analysis (PCA) and the analysis of the map covariance matrix have also played a highlighted role in the continuous heterogeneity analysis.^55,75,90,118,187^ In this case, the manifold is approximated by the linear space defined by the principal components. References (75, 90, and 187) are remarkable because the principal components were calculated directly from the images. However, ref (174) showed that a PCA performed considering only a few components is necessarily restricted to low-frequency motions. Another way of addressing the continuous heterogeneity problem is through normal modes analysis (NMA), as the HEMNMA algorithm showed.^53,72,167^ This algorithm studies the continuous conformational changes in the particles modeling the transition pathway with the help of NMA. Thus, it attempts to provide some light on the dynamics of the protein. To do that, it makes use of an atomic or pseudoatomic model of the macromolecule to predict the motions from the normal modes. The study of heterogeneous samples with normal modes was extended in a “local sense” by dividing the map into small regions and searching the combinations of normal modes that better explain the motion of the protein given the set of particles.^150^ Zernike3D^62^ can also estimate continuous deformations as NMA, but the deformation field is now continuously defined for all points in the space (not only at the center of the atoms or pseudoatoms), and it naturally introduces a coarse-to-fine movement decomposition that removes the need to manually choose the normal modes to explore. Given a continuous deformation field, ref (169) introduced a method to estimate the local rotations and strains by means of its differential analysis.

The last group of methods contains those that cannot be classified in the previous two groups. Multibody refinement^104^ assumes the flexibility of the macromolecule can be decomposed into independent rigid movements of structural regions, called bodies. Individual bodies can be solved at a higher resolution by masking the body region and isolating that region in the particle projection. These new particle images are then refined in the standard way, and the refined bodies are placed back into their original location within the macromolecule. A similar idea was proposed a few years before as proof of concept, named localized-optimization.^155^ This idea can be considered the natural evolution of the localized 3D classification.^9^ cisTEM and FREALIGN also introduced a 3D classification based on this concept of masking the region of interest to solve the heterogeneity and refinement problems.^223^ Klaholz^80^ proposed to perform multivariate statistical analysis of specific map regions.

Reference (165) introduced an interesting idea that can be considered to lie in between discrete and continuous heterogeneity analysis. Let us assume that we have a number of maps found by a discrete heterogeneity analysis. We may arrange them in a continuous low-dimensional map according to their relative distance. In this way, we may identify continuous trajectories followed by the macromolecular complex, helping in its dynamic characterization. This idea was further pursued in ref (62) with the combination of multiple criteria to perform the mapping to the low-dimensional space.

**Figure 15:**
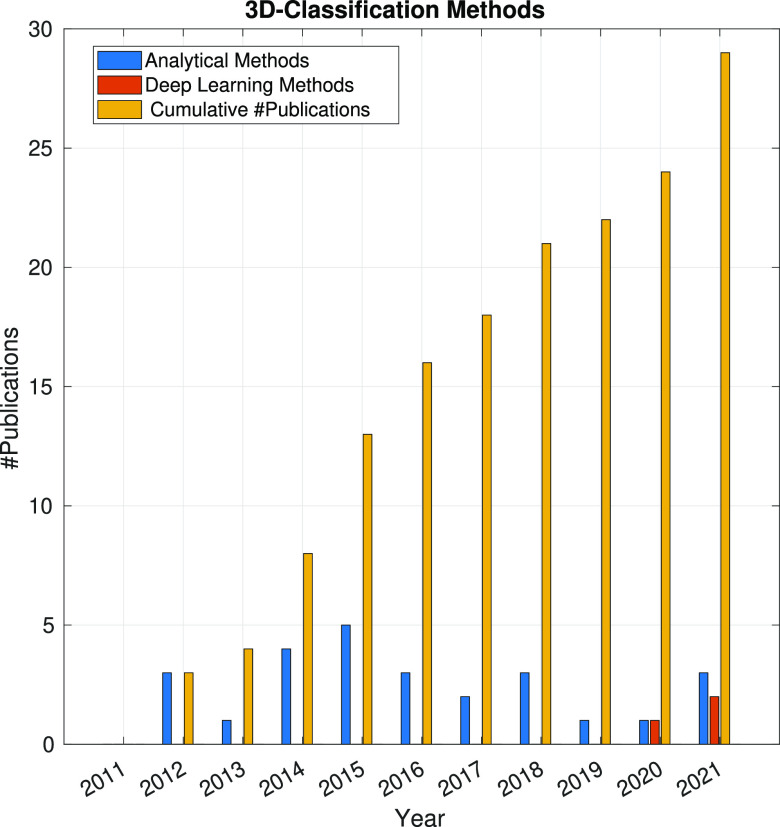
Time evolution of the number of publications about 3D classification based on analytical approaches or deep learning methods. The symbol #publications denotes the number of publications.

Figure 15 shows the time evolution of the number of publications related to the refinement step. We can see how the 3D classification step has been a constant topic of interest during the past decade. The reason is the critical biomedical information that specimen flexibility provides. In this way, methods have tried and still try to overcome the possibility of getting trapped in local minima. The analysis of continuous heterogeneity in the manifold framework seems a topic of high activity of research with promising results, although the problem of local minima is even more severe due to the larger number of parameters. We expect more developments in this regard during the next years. Moreover, due to the nonlinearity of the mapping onto a manifold, deep learning has started to play an important role in the definition of latent spaces.

## 11 Map Refinement

### 11.1 The Refinement Problem

The refinement step makes use of all particles assumed to belong to a given 3D class, meaning that the flexibility/heterogeneity problem (if it existed) is assumed to be solved and all particles are projections of the same conformation of the macromolecule. With all these particles, the map refinement step determines the relative orientation of the particles with respect to a reference volume. This process is iterative: starting from a reference volume, all particles are assigned an orientation with respect to it, and then the volume is updated using the experimental images and their orientations. The methods of projection matching and maximum likelihood explained in section 2.1 are the most common approaches for the refinement. Once the map is obtained after each iteration, the result is filtered according to its estimated resolution (see section 13), which determines the highest reliable frequency of the map over the noise level. This filtering prevents noise features from serving as anchors biasing the particle orientation estimation. Some other approaches, such as nonuniform refinement (explained later), apply a local filter according to the SNR, and in general, any postprocessing procedure that can identify and attenuate noise features can be employed. Finally, some minor refinements are carried out after angular refinement, such as better frame alignment, local CTF estimation and correction per particle, or the Ewald sphere correction. In general, all these steps are known in the field as polishing. The combination of these minor corrections allows for further pushing the quality of the reconstructed map.

One of the problems of this step is that we will always obtain a map as a final result. However, there is no guarantee that the obtained map is correct. The reason is that there are multiple optimizations along the path, and our refinement can be trapped into a local minimum far from the global representative solution. If this is the case, the resulting map will not represent the structure we try to solve, and any interpretation of its biological informational content will be wrong. This situation is known in the field as overfitting, and in ref (168) we show that this is caused by bias in the estimation of the various parameters involved in the image processing.

### 11.2 Refinement Methods

We can group the refinement methods in two categories: first, those methods that purely address the issue of angular assignment and reconstruction from projections; second, those methods aimed at enhancing the quality of the reconstructed structure (i.e., polishing).

- Group 1. Probably the most popular approach in the field is a Bayesian formulation of the angular assignment problem introduced by Scheres.^145^ The key idea behind the maximum *a posteriori* approach is to find the map that maximizes the likelihood of observing the experimental data at hand given some prior distribution of the maps being reconstructed. The prior is a Gaussian distribution of the Fourier components of the signal. A key component of its success was its implementation through the expectation-maximization method and the use of massively parallel hardware (GPUs).^78^ This method became dominant in CryoEM.
  A revolutionary approach appeared in 2017 with CryoSPARC.^119^ It introduced a combination of stochastic gradient descent and branch-and-bound approaches to solving the previously explained expectation-maximization problem.^145^ The combination of these two methods, along with good implementation and the use of GPUs, allowed for the refinement of high-resolution reconstructions in really short times. The stochastic gradient descent considers only random subsets of particles in each iteration, which reduces the complexity of the problem and avoids falling into local minima. The branch-and-bound approach speeds up the angular assignment by establishing a bound that prevents uninteresting regions of the parameter space from being explored.
  HighRes^176^ introduced the idea of removing nonsignificant features of the reconstructed map. A multiresolution approach is used to speed up the computational time and reduce the probability of getting trapped in local minima. Global search of the projection directions is promoted until the angular assignment is stable, and then a local refinement is performed.
  The nonuniform refinement method proposed by ref (120) follows the idea of filtering out those map features that cannot be reproduced in both halves of the data set. This filter is local, as the SNR is, in general, not uniformly distributed in space. This is particularly true for membrane proteins or heterogeneous samples.
  In the domain of deep learning algorithms, Jiménez-Moreno et al.^71^ introduced an algorithm in which an ensemble of neural networks determined particle orientation. Each network was responsible for recognizing the particles coming from a given orientation. Gupta et al.^51,52^ formulated the 3D refinement problem as a GAN problem. Experimental images are not explicitly assigned an orientation. Instead, a volume is reconstructed such that its projections cannot be distinguished from the experimental images by a discriminator network. This is one of the first works in which the physics of the forward image formation model and a neural network are jointly used in a single algorithm.
- Group 2. The idea of going back to the frames after a first reconstruction is obtained to fine-tune the image parameters was first proposed by Scheres^144^ as part of a motion correction algorithm that was discussed in section 4. The goal was to refine the BIM on a per-particle-per-frame basis, and later on, summing up all particle frames with some weights resulting in a new particle image called a *polished particle.* The weights aim at taking into account the radiation damage and its associated loss of information at high frequency. To that end, the ratio between the Fourier decay of the amplitudes of consecutive frames served as an estimate of the radiation damage. Zivanov et al.^233^ introduced Bayesian polishing as an extension of ref (144) but with a different way of estimating the relative amplitudes. In this case, they use the Fourier cylinder correlation (FCC), which measures the correlation between each particle frame and the reference at different frequencies. Thus, it is possible to minimize the distance between the FCC and an exponential model with a parameter that describes the weights of the frames to be summed during the polishing. The idea of weighting the different frames was also explored by Grant^50^ considering the electron dose and optimizing the contrast based on the SNR or in ref (12) taking into account the similarity between the frame content and the reconstructed map.
  The estimation of local defocus and high-order aberrations were the next refinement. In section 5 we have shown that the CTF is first estimated per micrograph. However, particles can lie at different heights within the sample, implying a different defocus per particle.^12^ Images are not only affected by defocus and astigmatism, but other aberrations also contribute to the loss of quality. To estimate these undesired effects on a per-particle basis, the reconstruction of the macromolecule must present a high resolution. The methods published in refs (234 and 235) refine the phase argument of the CTF by using Zernike polynomials that are an orthogonal basis of functions to describe surfaces on the unit circle. In all cases, this fine-tuning of image parameters has improved the resolution of the reconstructed map.
  Going ahead with modeling corrections, we encounter the effect of the so-called Ewald sphere. In CryoEM, the sample is intentionally defocused to increase the contrast to visualize the macromolecules in the sample. Furthermore, CTF models are based on the weak phase approximation that assumes elastic interaction between electrons and sample and a thin sample. When the sample is thick, these hypotheses are broken, and therefore, the CTF model is not fully valid. The main reason is that the weak phase approximation assumes a single defocus value or, equivalently, that the limited depth of field can be neglected. This assumption is generally correct for low frequencies, and all rays reach approximately the same image plane on the detector. However, for higher frequencies, the wave fronts start to be focused on different planes. The importance of the effect is resolution-dependent, with large macromolecules as the first candidates for enhancement.^36^ Several methods were proposed to correct the Ewald sphere curvature, such as the single-side-band CTF correction^139^ or the more recent ref (22). Experimental results agree with the considerations above, showing a clear improvement, especially in thick samples such as viruses.^188,231^

Despite all the advances in map refinement, we must remember that all these angular and imaging parameters have to be estimated in very noisy images. The estimation process can become rather unstable depending on the data set, and it is not uncommon that two different algorithms or two executions of the same algorithm disagree in their estimations for more than 50% of the particles in some unfavorable cases. Those incorrectly estimated parameters will necessarily bias the reconstructed map, an effect that is usually referred to as overfitting. This idea is further explained and experimentally validated in ref (168).

**Figure 16:**
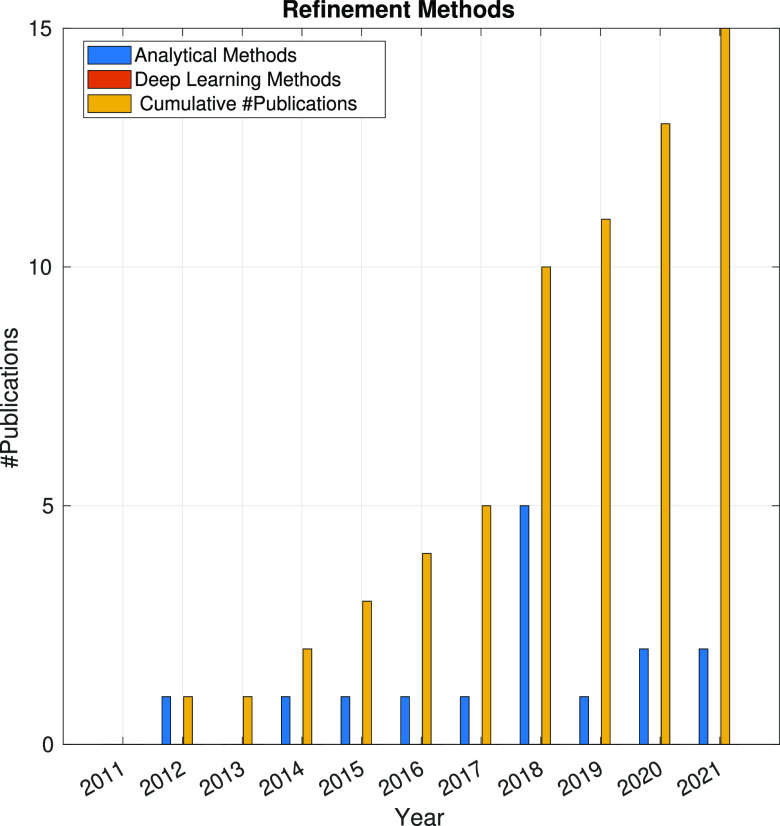
Time evolution of the number of publications about map refinement based on analytical approaches or deep learning methods. The symbol #publications denotes the number of publications.

Figure 16 shows the time evolution of the number of publications related to the map refinement step. We observe how the step of CryoEM refinement is evolving from the pure reconstruction algorithms that attempt to properly determine the angular assignment of the particle toward the introduction of physical constraints. The increasing amount of data available is stressing the current algorithms in two ways. First, the execution time must be kept within reasonable values without compromising the quality of the angular assignment. The second stress is imposing the need to choose or, at least, weight the different input images according to their quality and similarity to the conformation being reconstructed.

## 12 Validation

### 12.1 The Validation Problem

The reconstructed structure is not the end of the SPA workflow. Now, we want to determine if the obtained structure is reliable. The SPA workflow is composed of many steps, and the possibility of committing an error in any of them can result in a wrong reconstruction. In general, the main culprit of these errors is the low SNR of the images. Perhaps overfitting is the most widespread problem, and in many steps, such as angular assignment, the errors can result in low-quality or even incorrect maps. Although methods such as the gold standard (splitting randomly the set of particles in two subsets with half the images each, which are then independently processed to reconstruct two maps) are used to detect overfitting, they do not guarantee the absence of systematic errors committed in both data halves.^168^ Validation methods may analyze different features of the map itself, or for studies that propose an atomic model, they may consider the map and the structural model simultaneously. We should comment that early work on this topic included a report of a specially convened task force (the Validation Task Force), including a set of good practices to reduce the number of incorrect structures,^61^ and a review of some validation tools can be seen in ref (133).

### 12.2 Validation Methods

Validation methods were first considered by ref (134), where pairs of images at different tilts of the same specimen area were acquired. The idea of this work was that the geometry acquisition would impose two constraints, the angular orientation of the tilt pair of particles and the tilt angle and the tilt axis (rotation axis of the sample). Thus, by an independent search for the angular assignment of the untilted and tilted particles we could validate the alignment if the angular difference between them was given by a rotation of the tilt angle around the tilt axis. This difference was summarized in a polar plot in which the points corresponding to a correct angular assignment tended to cluster around a point related to the tilt angle. This work served as the theoretical basis of a validation server where the users could upload the particles and the reconstructed map and the server would produce the validation method mentioned above.^216^ Along these lines, Russo^137^ proposed a hypothesis test based on a Fisher distribution to quantify the clustering of the polar plot. The analysis of tilt pairs was also used to study the influence of the molecular mass in the angular alignment, as Henderson et al. showed.^60^ It showed that small molecules present a major uncertainty in their angular assignment. Tilt-pair validation is currently not much employed due to the use of cryo-samples and their low contrast in tilted images. Vargas et al.^201^ proposed another way of characterizing the angular assignment by studying the clusterability of the most similar projection directions (not only the best one, as is normally chosen by projection matching). A lack of clusterability reveals an intrinsic difficulty in aligning a set of images to a particular reference volume.

Another common issue related to validation is the existence of overfitting. This fact makes it so that noise can be reinforced in the alignment step resulting in an overestimated resolution. Chen et al.^24^ proposed a phase randomization of the particle images beyond a given frequency to detect overfitting. Moreover, they derived a formula to calculate the unbiased Fourier shell correlation (FSC; the FSC is a resolution measurement; see section 13). Also with the aim of detecting overfitting, Heynmann^63,64^ suggested the use of pure noise particle data sets of the same size as the sets used for reconstruction. The resolution achieved with a set of true particles of a given size should always be better. The size of the data sets was varied from small subsets to a final set with the same size as the total number of particles available. Along a similar line of reasoning, we have ResLog plot;^182^ the idea is to track the progress of the resolution as a function of the number of particles used. This curve can inform whether a particular study is limited by the number of particles or by other experimental factors such as intrinsic variability, difficulties in the alignment, etc.

A different approach to the validation problem was proposed by Cossio et al.^25^ This work complements the gold-standard technique by taking out a small subset of particles as the test set during the refinement. It allows for determining the probability of the refined map at each frequency given the test set. This probability should grow with the resolution in each refinement iteration and, therefore, discriminate between well-reconstructed maps and those obtained from noisy or empty particles.

A large group of validation methods compares the CryoEM structure with other determinations of the same structure with different experimental techniques such as X-ray,^127^ SAXS,^70,77^ or ion mobility mass spectrometry.^31^ Despite their interest, we feel that these techniques fall far from the image processing scope of this review.

**Figure 17:**
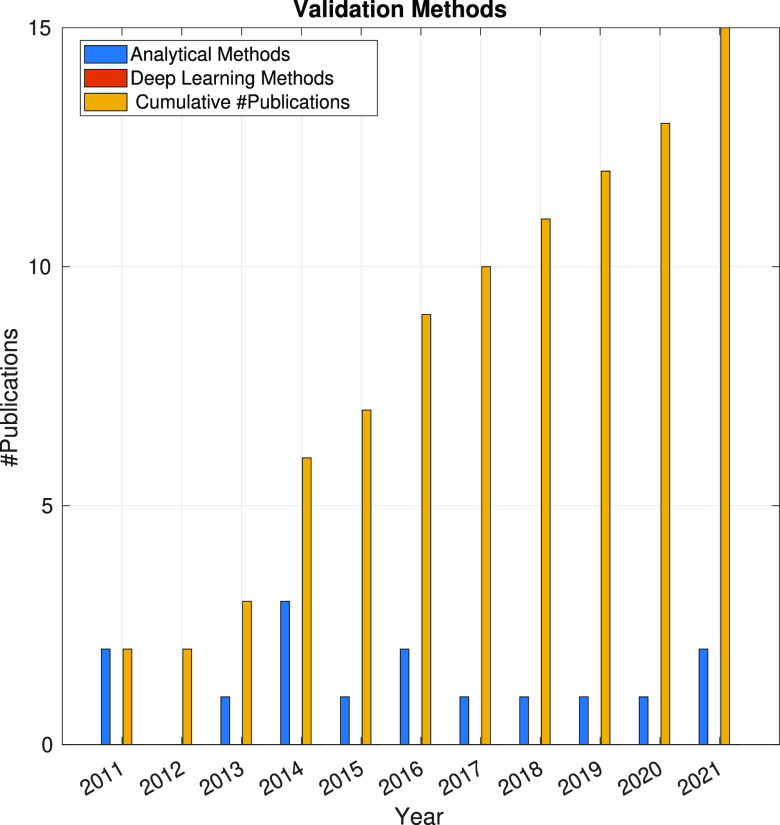
Time evolution of the number of validation-related methods based on analytical approaches or deep learning methods. The symbol #publications denotes the number of publications.

Figure 17 shows the time evolution of the number of validation-related publications. The validation methods have evolved from the compatibility or consistency of the raw data with the reconstructed map and the analysis of possible overfitting to validation of the atomic models traced from the reconstructed density maps. Despite this transition, issues such as alignability or the angular assignment are still critical because the finest details of the reconstruction are sensitive to problems in this task.

## 13 Resolution Analysis

### 13.1 The Resolution Problem

Every time we measure a physical magnitude, it is necessary to report its uncertainty or degree of reliability. Resolution analysis attempts to address this problem of estimating the degree of spatial reliability that a reconstruction presents. Even though the CryoEM community lacks a universal definition of resolution, it is widely defined as the size of the smallest reliable detail of the structure and, therefore, will be measured in length units, generally angstroms (Å). The resolution analysis can be global, when we try to analyze the quality of the whole map, or local, when a specific region is analyzed. The Fourier shell correlation (FSC)^54^ is the current standard for global resolution. It measures the cross-correlation between two half maps (i.e., two independent reconstructions using for each half the set of particles, followed by gold standard reconstruction) band pass filtered at different resolution shells. When a mask is provided to exclude the noise from the half maps, then the resolution changes due to the convolution in Fourier space implied by the mask. For a review of global resolution methods, see ref (171). The concept of resolution can also be extended into a directional resolution^189^ to determine if a given reconstruction is isotropic or anisotropic. Experiments with preferred orientations result in anisotropic reconstructions (the overrepresented directions have a larger SNR). Current global anisotropy metrics are the sphericity and the Fourier shell occupancy.^189,205^

In this way, we see not only that the resolution is a single number with which we may qualify the goodness of a reconstruction but also that it depends on the specific location and direction we consider (technically, this is called a tensor). In general, local and directional resolution values are better understood as relative “quality” descriptors between regions of the macromolecules, which may be affected in different ways by flexibility or compositional heterogeneity, besides errors in angular assignment. Additionally, we must emphasize that having a given global resolution is not a necessary condition to visualize some structural details (e.g., a resolution of 5 Å does not guarantee visualization of α-helices), but the visualization of some structural details implies given resolutions (e.g., the visualization of α-helices implies a resolution > 5 Å). The same argument could have been made with side chains, which should start to be visible at a resolution ∼ 3 Å.^204^ Finally, we would like to highlight that resolution analysis should be carried out with the raw reconstructed half maps without postprocessing.

### 13.2 Resolution Methods

In general, all resolution measurements are based on the gold standard method,^147^ that splits the set of particles into two independent subsets resulting in two independent reconstructions or half maps.

Concerning global resolution, in the past decade, there have been very few contributions. Reference (220) introduced *SRes* based on the spectral SNR and multiscale spectral analysis. Despite the existence of alternatives, the FSC remains the current standard, and in a way, this facilitates the comparison of the reported resolutions among different studies.

In the last ten years, there have been several new developments in local resolution. The first approach appeared in 2013 with blocres.^21^ This method considered a local FSC computed in a small window centered at the voxel of interest. By moving the window, the local FSC was computed for all voxels of the protein. Almost simultaneously, another method, ResMap, was also published to quantify the local quality of the reconstruction.^82^ However, the approach was completely different. ResMap employed a basis of steerable functions based on Hermitian polynomials that were used to approximate local sinusoidals. The resolution was then estimated with a hypothesis test to determine if the local sinusoidal fitted to the density was significantly above the noise level. A new approach was proposed in 2018, MonoRes.^207^ This algorithm estimates the local resolution by establishing a hypothesis test between the local amplitude of the signal (coming from the macromolecule structure) and the noise level of the map. This comparison was carried out at each frequency, and the resolution at which the local signal could not be detected above the noise (in a statistical sense) was defined as the local resolution value. To have access to local amplitudes, ref (207) makes use of the so-called monogenic signals. This method was also extended to electron tomography. References (124) and (208) introduced a local resolution approach based on deep learning, DeepRes. A neural network was trained with atomic models converted into density maps and band pass filtered at different resolutions. The network was then used to identify textures similar to those used during training and, therefore, infer the map resolution. Another deep learning approach was proposed by Avramov et al.,^7^ where neural networks were used to classify features according to their resolution, validating the resolutions on experimental reconstructions. In general, it is important to mention that different local resolution methods may produce (and often do) somewhat different estimations. The reason is that each of these methods considers a different property to define the resolution and even the very notion of “locality”, as is discussed in ref (204) in a work addressing good practices for local resolution estimation. Other conclusions of the latter work are that local resolution should be estimated from raw half maps only, without any postprocessing or sharpening (except with DeepRes, which is based on textures).

After tackling the estimation of local resolution, the field addressed the problem of resolution anisotropy. The existence of preferred directions reinforces the signal along the planes perpendicular to the preferred directions of the particles. In 2017, Tan et al.^189^ proposed that the existence of preferred directions could be alleviated by tilting the sample. To show that, they calculated a directional FSC considering a cone of 20 degrees as a directional filter, obtaining the so-called 3DFSC (defined as the isosurface of the directional FSCs along all possible directions). Thus, the sphericity of the 3DFSC was proposed to evaluate resolution anisotropy. The closer to a sphere, the more isotropic the reconstruction is. Related to this work and almost simultaneously published, Naydenova and Russo^107^ addressed the issue of how preferred orientations affected the quality of the map and proposed a way to estimate anisotropy based on efficiency, a statistical parameter that characterizes the orientation distribution with a point-spread function. This work also showed the importance of tilting the sample to alleviate problems related to preferred directions. MonoDir^209^ extended the method of MonoRes to measure local and directional resolutions. As in MonoRes, resolution is measured through a hypothesis test on the energy of the local amplitude at different frequencies. The difference is that this measurement is performed on a filter bank of directional filters. Recently, a very simple metric for the simultaneous estimation of the FSC-resolution and anisotropy was proposed, the Fourier shell occupancy (FSO).^205^ The FSO informs about the percentage of information at each resolution (Fourier shell) compared to the FSC shell, showing that resolution anisotropy cannot be reduced to a single number; that is, anisotropy is a spectral property. The authors prove that the value FSO = 0.5 occurs exactly at the FSC resolution. Thus, the article addresses the simultaneous measurement of global resolution and global anisotropy and provides a mathematical formalism for directional filtering and understanding the statistical behavior of both the FSC and FSO. The induced resolution anisotropy as a consequence of the particle direction distribution was also studied in the latter work, showing that resolution is certainly affected by the particles’ orientations; in other words, the resolution is affected by the sampling of directions, and anisotropy can be considered a consequence of a nonuniform sampling. The sampling compensation factor (SCF) was introduced to characterize the effect of the angular sampling on the SSNR (spectral signal-to-noise ratio).^10,11^

The last group of research topics related to the resolution step addresses the effect of different masks on estimating the resolution and its uncertainty. Two recent works have addressed these topics. The first one, the mFSC,^114^ proposed to invert the order of application of the mask in the FSC estimation; more precisely, instead of masking and computing the cross-correlation of the masked maps at different frequencies, this method proposed to band pass filter the half maps and mask them to compute the cross-correlation. This strategy alleviates the effect of the mask on the FSC but considerably increases the computational burden. The same work also analyses the FSC error using Fisher’s *z* transform, providing a confidence interval for the resolution estimation. The second approach, by Beckers et al.,^14^ proposes random permutations of the Fourier shells in the half maps. This allows for estimating multiple FSCs to determine the FSC distribution and, therefore, to infer a confidence interval for the FSC (resolution error). In addition, this approach seems to be stable under different mask geometries.

**Figure 18:**
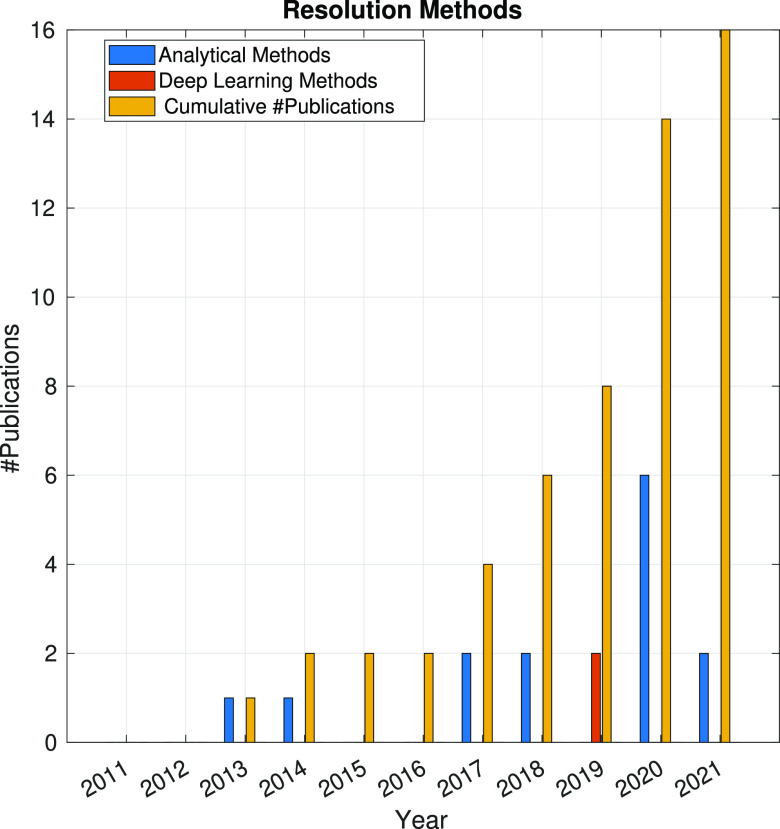
Time evolution of the number of publications of resolution-related methods based on analytical approaches or deep learning methods. The symbol #publications denotes the number of publications.

It is interesting to note how an old topic like resolution estimation has been and still is an issue of sustained and varied work. Traditionally, the controversy about the FSC threshold has always been present.^171^ Still, the last 10 years have witnessed the emergence of local resolution, local-directional resolution, resolution anisotropy, resolution error, and mask dependency. Figure 18 shows the time evolution of the number of publications related to resolution estimation. The use of deep learning methods is starting in the resolution field, so that most of the publications in this regard correspond to analytical approaches. This can be explained partly due to the relative novelty of deep learning approaches and partly because of the desire to root these metrics into a defined statistical signal processing background, which can be difficult to achieve with deep learning.

## 14 Volume Restoration

### 14.1 The Restoration Problem

Understanding the biological behavior of a given macromolecule is one of the goals of structural biology. The reconstructed map contains the spatial information about the macromolecule, and from it, we would like to interpret it in terms of an atomic model. However, this information can be complex to analyze. For instance, reconstructed maps are usually visualized by establishing a threshold that defines an isosurface; thus, only densities greater than or equal to the threshold are shown. This representation can be suboptimal because it depends on an arbitrarily chosen parameter, the density threshold. We can find reconstructions for which some connections between densities do not have enough contrast in the map and are, therefore, difficult to trace in atomic terms. In other situations, such as those corresponding to maps presenting regions of very different local resolutions, it is necessary to change the density threshold to enhance the visualization of one region over another. Thus, it would be very desirable to have a map transformation that enhances the visualization of the protein for its understanding, helping trace the amino acid chain. This is what sharpening methods do. This transformation normally involves a high-frequency boosting and a map denoising/masking. Special care must be taken to avoid oversharpening. Note that this boosting of the high-frequency components changes the spectral properties of the reconstruction in ways that may be rather complex so that the quantitative use of sharpened maps beyond visualization should be handled with great care.^206^

### 14.2 Restoration Methods

Sharpening algorithms can be grouped into two types: global and local sharpening methods. The most widespread method is the B-factor correction.^134^ It is a global transformation that carries out a flattening of the spectrum of the protein, taking into account the FSC. The idea is to get a sharp visualization of the high frequencies hidden by the low-resolution information. This is what RELION postprocessing does.^145^ AutoSharpen^192^ proposed to look for the best B-factor considering two objective functions simultaneously: the sharpened map must have a maximum connectivity and minimum surface. Leaving the weighting by the FSC out, the B-factor correction considers that the reconstructed map is the result of a convolution of a sharpened map with a Gaussian and isotropic PSF (point-spread function). This assumption is the starting point of many sharpening methods. For instance, Hirsch et al.^65^ used a blind deconvolution to determine the sharpen map considering some constraints such as non-negativity, smoothness, and sparseness of the map. Other approaches such as the one followed by Kishchenko et al.^79^ assume that the blurring of the protein is a consequence of inaccuracies in the angular assignment of the particles. Then, the introduced error must be purely tangential and will grow with the distance to the origin. They suggested a spherical deconvolution to restore the map. Another method called VISDEM^180^ makes use of the number of atoms that the protein has as a constraint. Normally, this information is known by means of other techniques, and if it is not, the method provides mechanisms to estimate it. Considering the number of atoms and the shape of the protein (obtained by thresholding), the volume is filled (coarse grain model) with pseudoatoms, and a refinement of the density distribution and radial spectrum is carried out. The coarse-graining technique was also used as a denoiser in ref (73).

Local sharpening methods started as a trend in 2017 with the algorithm of LocScale.^69^ The idea was to carry out the B-factor correction in a local sense to obtain a local spectrum similar to the local spectra of a reference atomic model, although the method can also internally handle other possibilities. LocalDeBlur^123^ addressed the problem of local sharpening as a local deconvolution where the local PSF depends on the local resolution of each voxel. This method has proven to be very effective when the maps present regions with very different resolutions. LAFTER^125^ makes use of two half maps to recover the part of the signal that is not buried in noise. To do that, the maps are band pass filtered at different resolutions, and the voxels of the band pass filtered maps are locally weighted, according to their probability of being signal and noise. Then, the weighted and filtered maps are added and an eighth-order Butterworth low-pass filter is applied. This method is the basis of SIDESPLITTER,^126^ where the map restoration step is integrated into the map refinement process. A similar approach was proposed by ref (76), only that the local energy by frequency is estimated using the spiral transform that can decompose a function as the product of an envelope and a phase. The details of the weighting used to reconstruct the sharpened map are also different. Finally, we comment on local density modification methods that incorporate prior knowledge coming from the atomic nature of the map being reconstructed. This approach introduced by Terwillinger et al. is very often used in crystallography and was recently introduced in CryoEM to improve the interpretation of the maps.^191,193^

All these restoration approaches are based on analytical methods, and we had to wait until 2021 to have the first deep learning sharpening method, DeepEMhancer.^141^ Indeed, the high number of CryoEM reconstructions with fitted atomic models already deposited at the Electron Microscopy Data Bank (EMDB) and PDB^16^ databases is enough to train a neural network. The neural network must learn the shape of atomic models converted into density maps from the shape of the reconstruction. At very high resolutions, there are not enough maps for the training, and deep learning algorithms may not be so useful.

**Figure 19:**
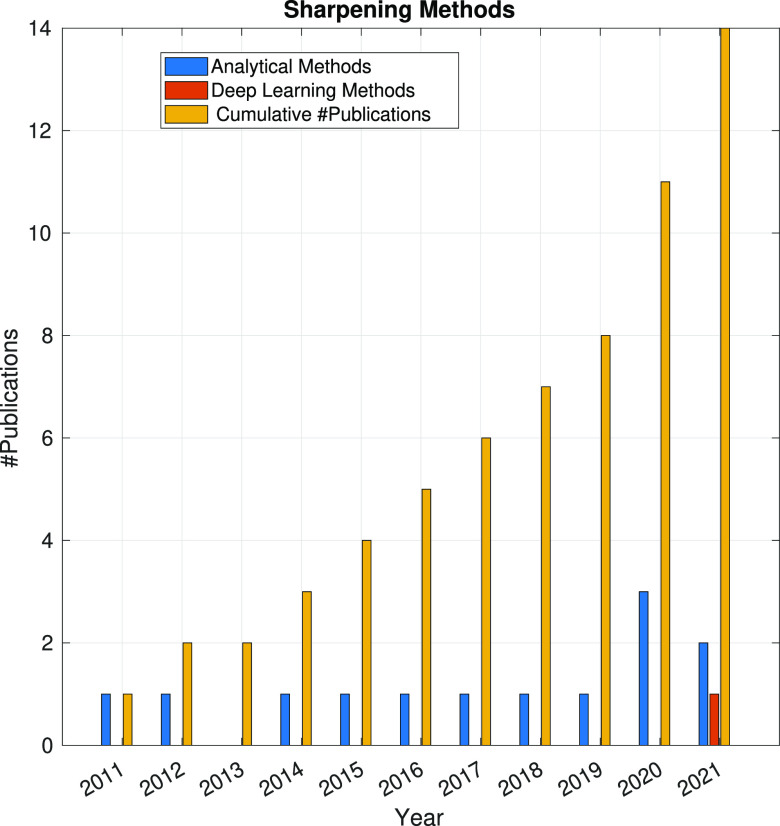
Time evolution of the number of publications on map restoration based on analytical approaches or deep learning methods. The symbol #publications denotes the number of publications.

Figure. 19 shows the time evolution of the number of publications related to map restoration. It can be observed that the majority of the methods published in the the last ten years are analytical. In the future, we expect the introduction of anisotropic approaches to sharpening, perhaps based on deep learning.

## 15 Remaining Problems and Emerging Topics and Methods in CryoEM

As we have seen throughout the paper, there have been many new image processing and data analysis methods in the past 10 years. In Table 1, we show a summary of these papers over the years and topics. From this table, we can see that there are three different topics trends depending on the number of new methods in each one of them. Smaller activity does not mean that a particular problem is unimportant or that it is solved. However, on the other hand, a large activity probably means that the problem is really at the core of the data analysis path and that the problem is still perceived as open either in its basic or more advanced aspects.

- Low-activity topics: Micrograph and particle denoising, together with map segmentation, are the three topics with the lowest activity in the last ten years. We note, however, that activity in these areas is also more recent, concentrating in the second half of the decade. Probably, micrograph and particle denoising are outside the main data analysis path. They are helpful as auxiliary steps to other tasks, such as particle picking or image alignment. These auxiliary tasks are not commonly used because the standard data analysis pipeline has already been designed to deal with the high levels of noise found in the raw data. Instead, map segmentation is normally performed by simple isosurface thresholding in the different visualization programs. Although more sophisticated approaches can be employed, these are useful for interpreting subtle details as loops, side chains, or other weak details.
- Medium-activity topics: We may identify two different trends here: those topics for which there was a larger activity in the first half of the decade and those for which a larger activity occurred in the second half.
  1. First half: In this category, we find movie alignment, CTF determination, initial volume, and map validation. This is logical, as these steps are crucial to settle the bases of obtaining a map by CryoEM.
  2. Second half: In this category, we find map refinement and restoration. Again, this is logical; once the bases are settled, we want to fine-tune the parameters to reach high resolution (refinement) and gain more details by postprocessing the map (restoration).
  Interestingly, 2D classification has equal contributions in both halves, meaning that new ideas regarding identifying images constantly appear.
- High-activity topics: These are the workhorse tasks of the data analysis pipeline: particle picking, 3D classification, and resolution. The relatively higher activity in these domains shows the healthy condition of the field and how new advances are steadily being adopted.

**Table 1:** Number of Papers per Year and Topic

Year/Step	Movie Alignment	CTF	Particle Picking	2D Class	Denoising	Reconstruction	Initial Volume	3D Class	Refinement	Segmentation	Validation	Resolution	Restoration	Total
2011	0	3	0	1	0	1	0	0	0	0	2	0	1	8
2012	1	1	0	1	1	1	1	3	1	0	0	0	1	11
2013	3	1	4	0	0	0	3	1	0	0	1	1	0	14
2014	1	1	1	1	0	0	1	4	1	0	3	1	1	15
2015	3	2	1	1	0	3	4	5	1	0	1	0	1	22
2016	0	1	1	2	3	0	2	3	1	0	2	0	1	16
2017	2	0	2	1	0	0	0	2	1	1	1	2	1	13
2018	0	0	3	1	1	0	2	3	5	0	1	2	1	19
2019	0	1	9	0	1	0	0	1	1	1	1	2	2	19
2020	1	2	1	2	1	0	0	2	2	2	1	6	3	23
2021	0	0	2	2	1	0	0	5	2	1	2	2	3	20
Total	11	12	24	12	8	5	13	29	15	5	15	16	15	180

In the following paragraphs, we comment on the current trends for each one of the topics, which are the most active areas of research for each one, and which are the problems that are still perceived as open:

- Movie alignment:
  1. One of the main problems here is the accuracy of the local alignment. It must be remembered that at, the frame level, the SNR is below 10^–3^ and that neither the signal nor the noise follow a Gaussian distribution (and consequently, tools such as Euclidean distance or cross-correlation are suboptimal). This accuracy is crucial to achieving very high resolution, but it does not need to be fully solved at the beginning of the image processing pipeline. Instead, it can be tackled at the end of the process in a step normally referred to as polishing,^12,233^ essentially fine-tuning the frame alignment parameters.
  2. Electron microscopes are continuously increasing their acquisition speed and the size of the recorded images. At present, movies can be acquired in <10 s, which is the time to perform the movie alignment if we are processing the data in streaming. This time constraint can be alleviated if we employ several GPUs in parallel, but in any case, there is a huge pressure on movie alignment algorithms to do their task in a very short time.
- CTF determination: Following the general trend, the processing speed and accuracy of the estimated parameters are still open problems in the field. Speed is normally addressed using GPUs. Regarding accuracy, this is normally not explicitly contemplated. However, to reach a very high resolution, the defocus must be determined with <100 Å of error.^226^
  The per-particle refinement of the defocus aims at filling this need for accurate defoci. The same can be said of the handling of tilted samples, in which each region of the micrograph has a different defocus. Estimating high-order aberrations is a step further in modeling the transfer function experienced by each particle trying to model it to very high resolution. The accuracy of the CTF of each particle is, undoubtedly, an open problem in which more research should be expected.
- Particle picking: Despite quick advances in recent years, especially with the adoption of deep learning algorithms, particle picking will probably remain as one of those areas with constant progress and continuous appearance of new ideas. Current tools can handle large data sets within a reasonable computational burden. Still, in the best case, the number of false positives can easily be as high as 10%.^142,232^ In more difficult cases, false positives can rocket up to 60–70%; the possibility of introducing structural bias is also a concern. Some algorithms have been developed to compute a consensus of multiple pickings smartly and eliminate obvious contaminating particles. False negatives are currently not a problem, as we acquire thousands of micrographs containing several millions of particles and losing some of them is not a catastrophe. Open problems in this domain are (1) decreasing the number of false positives without introducing bias; (2) addressing difficult situations such as micrographs close to focus, very low-molecular-weight particles, picking in thick ice, or finding minority populations; and (3) automatic picking without any human intervention.
- Clustering in 2D: Classifying particles into homogeneous 2D clusters may be one of the most classical image processing problems, probably dating back to the 1970s. Current research focuses now on the following: (1) how to handle millions of particles efficiently and automatically; (2) how to divide the input data set, on the order of millions of particles, into many distinct groups avoiding the so-called attraction problem by which the classes with higher SNR get most of the input images even if their representative does not correspond to the underlying image; and (3) exploring different image similarity metrics, trying to make the classification more robust to outliers, contaminations, low SNR, etc.
- 3D angular assignment: The next three topics are intertwined. Still, we will try to give a separate view of each one of the issues. 3D angular assignment addresses the problem of finding the orientation of a set of particles with respect to a reference map (ideally, a map containing minimal information, or even none). The open problems and current research lines include the following: (1) trying to minimize the dependence on an incorrect reference map and looking for a global minimum for each one of the particles, including a way to assign quality metrics to the angular assignment on a per-particle basis; (2) trying to escape the attraction problem that also occurs in 3D (as one direction gets higher SNR, it attracts experimental images even if they do not come from that direction); and (3) how to solve the problem efficiently either by changing the algorithm or by implementing it in massively parallel hardware so that millions of images can be handled in a reasonable amount of time.
- 3D reconstruction: 3D reconstruction can be considered a regression problem in which we try to find a signal model, the map, that is compatible with the acquired data. As in any other regression problem, the open problems are as follows: (1) being robust to large amounts of noise through regularization, the use of a smooth basis that reduces the number of parameters to estimate, or the addition of constraints; (2) fully accounting for the image formation model (for instance, explicitly considering high-order effects, such as the Ewald sphere correction or high-order microscope aberrations), including even possible elastic deformations of the structure being reconstructed; (3) including a priori information about the kind of objects being reconstructed through suitable priors in a Bayesian setup; and (4) solving the problem efficiently, as millions of particles may be involved in this step.
- 3D classification: This process tries to identify structurally homogeneous populations of particles. As with the previous topics, current research lines address the following: (1) finding the global minimum of the goal function being optimized and (2) handling continuous heterogeneity accounting for the continuous flexibility of biological macromolecules.
- Resolution determination: The resolution of the reconstructed map is the most typically reported quality measure of the final result. However, this concept is ill-defined, as it could refer to optical resolution, SNR, the self-consistency of our data analysis, or the presence of structural details of a given size. The four aspects make sense, and there could be multiple ways of measuring each of them without agreeing with each other. What is important is that there is at least one common way of reporting the quality, and this has been achieved at the level of the entire field by reporting the resolution at which the FSC drops below 0.143. The specific number may not be so important, in general, but what is important is that collectively we report in the same way. Current research focuses on locally and directionally characterizing the SNR and removing the influence of the measuring mask in the reported values. The elucidation of which factors are responsible for the loss of resolution is still an open question, and these factors probably change from one experiment to another. Despite there being some advances, the CryoEM community lacks metrics to identify such problems. For instance, the existence of angular alignment errors can be measured with the local-directional resolution, while also giving some clues about the possible existence of preferred directions; however, there is still room for new methods.
- Map restoration: A common current practice is to postprocess the raw output of the 3D reconstruction process. This has been termed map sharpening, and the goal is to boost the weak high-frequency components of the reconstructed map to distinguish better small details such as side chains, loops, or even water molecules around our map. At the same time, we want to remove all possible spurious artifacts unrelated to our structure. Current research lines follow either a pure signal processing approach somehow exploiting the SNR or the incorporation of priors based on the knowledge of the structure at high frequency of the building blocks of biological macromolecules (atoms, amino acids, secondary structure, protein folds, ...).
- Map validation: Another vital topic is the verification of the correctness of the structure obtained. Current research tries to do so by the following: (1) verifying the self-consistency of the data analysis pipeline; (2) verifying the consistency of the reconstructed map to data that has not been used during the reconstruction; (3) trying to identify possible parameter misestimates, most importantly in 3D angular assignment and classification; (4) verifying the consistency of the reconstructed map to other biophysical measurements; and (5) verifying the correctness of the properties observed for biological macromolecules at the given resolution.
- Atomic model fitting: The ultimate goal of a structural study with CryoEM is to elucidate the location in space of the atoms of the macromolecule. Since we currently reach a high-resolution map in many cases, the last step is usually the fitting of an atomic model to the reconstructed map. Current research lines try to do the following: (1) automate this process as much as possible, including rigid and flexible fitting; (2) avoid the local minima of the fitting goal function; (3) provide a measure of the uncertainty of the fitting (e.g., by producing an ensemble of models rather than a single structure); (4) extend these modeling capabilities to lower resolution maps; and (5) incorporate other biophysical measures such as those coming from mass spectroscopy, domain–domain interactions, or evolutionary constraints.

The whole image analysis pipeline in CryoEM can be regarded as a succession of small problems in which we need to estimate some parameters (the parameters describing the local movement of frames in movie alignment, the defocus parameters in the CTF estimation, the location of a particle in particle picking, etc.). These parameters themselves can be considered to be random variables, and in such a noisy environment, all these estimates are expected to be rather noisy. Our estimate can be relatively close to the underlying ground truth or rather far away. However, with a single parameter estimation, it is impossible to know which situation we are in. The only way to know is by estimating the same parameter multiple times and comparing the different outcomes. This is a rare practice in the field, but current research is heading toward calculating so-called consensus parameters (parameters that are consistently estimated in the same region). Only for these stable parameter estimations can we be more certain that we have successfully found a more or less correct parameter. On the opposite side, just taking the output of a single execution of any of the algorithms involved leaves us in a fragile position from a statistical point of view.

## 16 Conclusions

**Figure 20:**
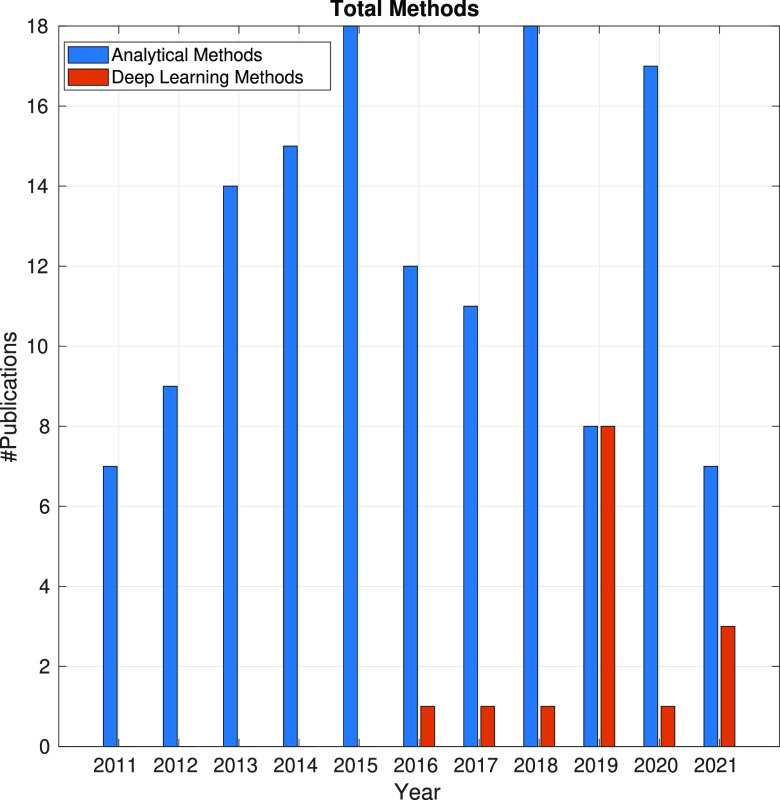
Time evolution of the number of publications on all workflow steps.

After observing what has happened in the last 10 years, we may draw some interesting conclusions:

1. There is no doubt that the resolution revolution in recent years has come from advances on multiple fronts: improvements of the reproducibility of sample preparation, the introduction and further advancement of direct electron detector cameras, improvements in the stability, automation, and better electron optics at the electron microscope, and the development of faster and more robust image processing and data analysis methods, which is the topic of this review.
2. The average number of new methods per year and topic is about 1.5, which gives a total of 15 new methods per year. These numbers show the healthy and active condition of the field. Additionally, the number of methods in the second half of the past decade is larger than that in the first half, pointing to acceleration and incorporation of new engineering or physics groups into the field. This steep increase in new methods makes it difficult for users to keep up the pace, especially if they have to change from one platform to another to use them. In that regard, an integrative platform where most of them are accessible is indeed handy not only because it simplifies their use but also because it allows for comparing the results from the different software tools solving the same problem. This comparison allows the scientist to find which parameters can be relied on and which are less reliable.
3. About 10% of the algorithms come from deep learning (see Figure 20), and most of them appeared in the second half of the decade. The problems in which these algorithms have mostly appeared are the expected ones: particle picking, map sharpening, validation, and tracing. However, they are starting to appear in more core tasks such as 3D reconstruction, angular assignment, and classification. In the coming years, deep learning algorithms will probably fully erupt onto the scene, and very likely, we will see hybrid algorithms and a more accurate consideration of the image formation model underlying CryoEM data acquisition.
4. Still, classical methods have advantages over deep learning approaches, which are seen as black boxes: (1) they allow explicit modeling of the physics and close understanding of the underlying mechanisms; (2) even though we now have millions of images, deep learning algorithms require labeled data; in this aspect, classical algorithms have a clear advantage, as they can work with very few images as well.
5. We may recognize two main trends in the development of new methods: (1) addressing more subtle details (“high-order”) or more difficult (lower molecular weight, lower contrast, continuous flexibility, etc.) problems and (2) decreasing the user dependence through the incorporation of automatic, smart decisions based on the data itself.
6. Although it is not common among practitioners, from the algorithmic point of view it has been recognized that the parameters required along the image processing pipeline are extremely noisy and that the fraction that can be reliably determined can be as low as 20% in some difficult projects and between 50 and 70% in more standard projects. The fraction of incorrect parameters (incorrect angular assignment, incorrect 3D class, incorrect particle, incorrect defocus, ...) is biasing our result. Consensus algorithms are being introduced to identify these situations, and in the future more algorithms of this kind should be expected.
7. Over the past decade, we have witnessed an increase from a few tens of thousands of particles at the beginning of the decade to a few millions at the end. This has put a formidable pressure on computational performance, and currently, the most successful algorithms invariably require GPU acceleration.

Overall, we are experiencing a sweet moment for technical advances in this discipline. The increased automation, robustness, and smart algorithms are shifting the image processing and data analysis in single particle analysis from art to routine. This can be seen by the quickly increasing number of maps deposited at the EMDB, many of them coming from groups that have recently adopted CryoEM as one more experimental technique within their reach.
